# Uncertainty-Dependent Extinction of Fear Memory in an Amygdala-mPFC Neural Circuit Model

**DOI:** 10.1371/journal.pcbi.1005099

**Published:** 2016-09-12

**Authors:** Yuzhe Li, Ken Nakae, Shin Ishii, Honda Naoki

**Affiliations:** 1 Graduate School of Biostudies, Kyoto University, Kyoto, Japan; 2 Graduate School of Informatics, Kyoto University, Kyoto, Japan; 3 Imaging Platform of Spatio-temporal Information, Graduate School of Medicine, Kyoto University, Kyoto, Japan; Harvard University, UNITED STATES

## Abstract

Uncertainty of fear conditioning is crucial for the acquisition and extinction of fear memory. Fear memory acquired through partial pairings of a conditioned stimulus (CS) and an unconditioned stimulus (US) is more resistant to extinction than that acquired through full pairings; this effect is known as the partial reinforcement extinction effect (PREE). Although the PREE has been explained by psychological theories, the neural mechanisms underlying the PREE remain largely unclear. Here, we developed a neural circuit model based on three distinct types of neurons (fear, persistent and extinction neurons) in the amygdala and medial prefrontal cortex (mPFC). In the model, the fear, persistent and extinction neurons encode predictions of net severity, of unconditioned stimulus (US) intensity, and of net safety, respectively. Our simulation successfully reproduces the PREE. We revealed that unpredictability of the US during extinction was represented by the combined responses of the three types of neurons, which are critical for the PREE. In addition, we extended the model to include amygdala subregions and the mPFC to address a recent finding that the ventral mPFC (vmPFC) is required for consolidating extinction memory but not for memory retrieval. Furthermore, model simulations led us to propose a novel procedure to enhance extinction learning through re-conditioning with a stronger US; strengthened fear memory up-regulates the extinction neuron, which, in turn, further inhibits the fear neuron during re-extinction. Thus, our models increased the understanding of the functional roles of the amygdala and vmPFC in the processing of uncertainty in fear conditioning and extinction.

## Author Summary

Animals live in environments that contain uncertainty. To adapt to uncertain situations, they flexibly learn to associate environmental cues with rewards and punishments. Understanding how the brain processes uncertainty has remained an important issue in neuroscience. To address this question, we focused on neural processing in the amygdala and mPFC during fear conditioning and extinction. We developed a neural circuit model that incorporates distinct neural populations in the amygdala and mPFC. Our model first successfully reproduced uncertainty-dependent resistance to the extinction of fear memory. An extension of the model provided a possible explanation for observations made during optogenetic manipulation of the ventral mPFC. Finally, we proposed a procedure to accelerate the efficacy of subsequent extinction based on our model.

## Introduction

**Fig 1:**
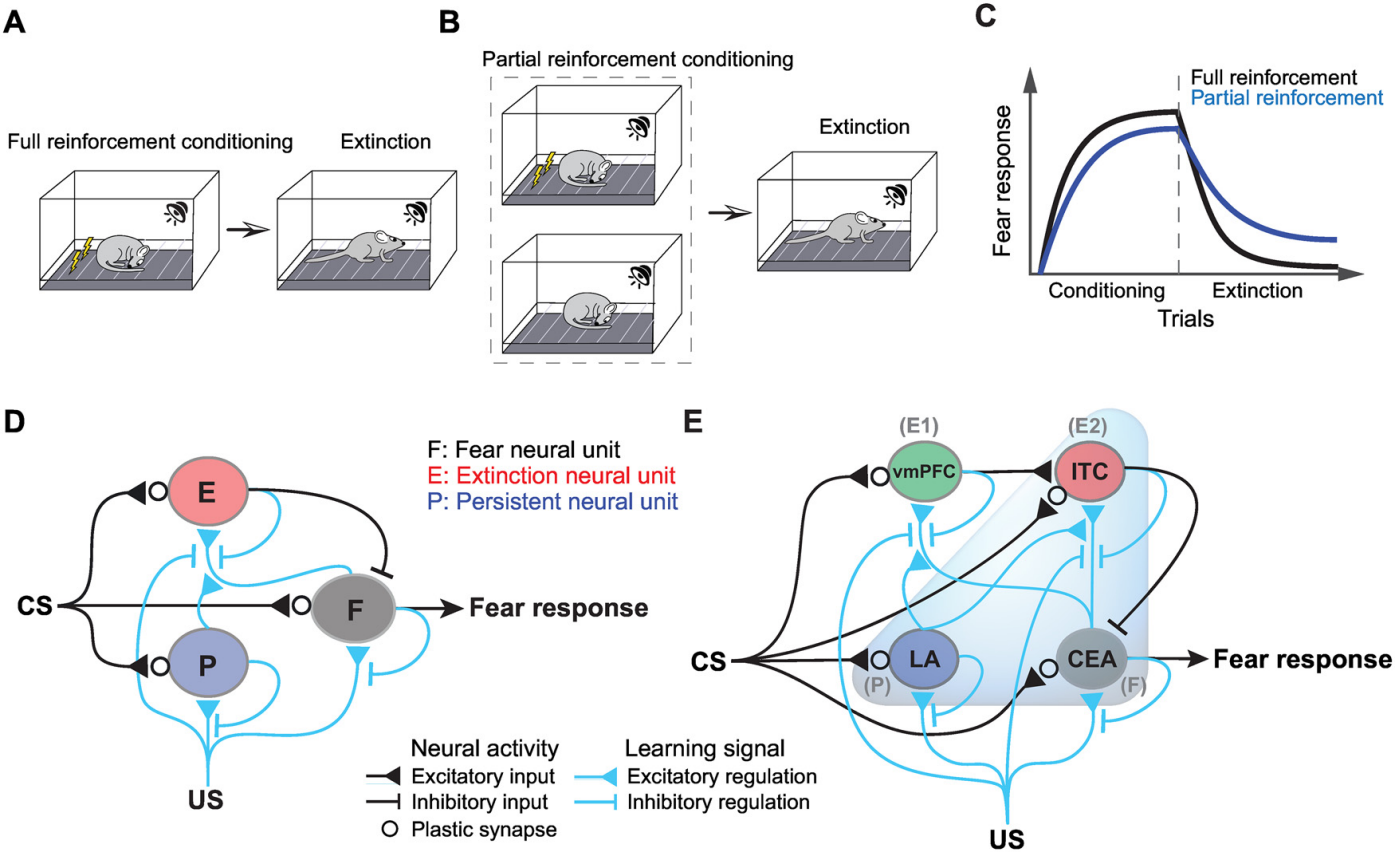
Partial reinforcement effect and the neural circuit models. **(A, B)** During fear conditioning, a CS, *e*.*g*., a tone, was fully (in the full reinforcement schedule **(A)**) or partially (in the partial reinforcement schedule **(B)**) paired several times with a US, *e*.*g*., electric foot shock (**left panels**). The fear memory formed during fear conditioning can be diminished by extinction training, during which the CS is repeatedly presented alone, without the US (**right panels**). **(C)** Conditioned responses to the CS, which are usually measured as the degree of behavioral freezing responses, are depicted during fear conditioning and extinction. The fear memory (measured as the degree of behavioral freezing responses) that was acquired through the partial reinforcement schedule with P(US|CS)<1 exhibits a PREE (**blue line**), which is evident as increased resistance to extinction compared to that of the fear memory acquired through the full reinforcement schedule with P(US|CS) = 1 (**black line**). **(D, E)** The two neural circuit models are shown as schematics. Black and blue lines describe synaptic connections and the learning signals that regulate plasticity at synapses indicated by black open circles, respectively. **(D)** The basic model based on fear, persistent and extinction neural units (F, P and E). CS-related input activates all the units, and the extinction neural unit inhibits the fear neural unit, the activity of which represents the strength of the fear memory (black lines) (eqs (1–3)). The efficacy of CS-related input to the fear, persistent and extinction neural units changes based on the learning signals (blue lines) (eqs (4–6)). **(E)** Extended model including subregions of the amygdala (the LA, CEA and ITC) as well as the vmPFC. In this model, the LA and CEA correspond to persistent and fear neural units, respectively, and there are two extinction neural units: the ITC and vmPFC. CS-related input activates all subregions, and the ITC receives excitatory input from the vmPFC and inhibits the CEA (black lines) (eqs (7–10)). A behavioral fear response was triggered by the CEA. The efficacy of plastic synapses (black open circles) changed based on learning signals (blue lines) (eqs (11–14)). Parameter values are listed in S1 Table.

The associative memories acquired through both appetitive and aversive conditioning with uncertainty have been shown to exhibit substantial resistance to extinction, known as the “partial reinforcement extinction effect (PREE)” [1–3]. For example, the fear memory acquired through a fear conditioning procedure in which the CS is probabilistically paired with the US (partial reinforcement) is more resistant to extinction than the fear memory acquired after full pairings of the CS and US (full reinforcement) (Fig 1A–1C). This initially sounds paradoxical because one may assume that the causal relationship between the CS and US is strong and weak in the cases of full and partial reinforcement, respectively [4]. Although psychological theories [5–7] and computational models [8–12] for how uncertainty induces paradoxical PREE have been proposed, the neural underpinnings of the PREE remain largely unclear.

The neural substrates implicated in fear conditioning and extinction are the amygdala and mPFC, respectively. The amygdala is a major region for the acquisition and expression of fear memory [13–15]. In contrast, the ventral subdivision of the mPFC (vmPFC), called the infralimbic cortex (IL) in rodents and the ventral mPFC in primates [16], plays an important role in the extinction of fear memory [17–19]. Although both the amygdala and mPFC function during partial reinforcement fear conditioning [20–25], their roles in the PREE have rarely been examined [26]. Thus, how the amygdala and mPFC are coordinated for the PREE remains elusive.

Recently, the electrophysiological properties of neurons in the amygdala and mPFC have been extensively investigated; interestingly, three different types of neurons have been identified and defined as the following basic properties: “*fear neurons*”, which exhibit CS-evoked activity (spike firing) after fear conditioning and abolished activity after subsequent extinction; extinction-resistant “*persistent neurons*”, which also exhibit CS-evoked activity after fear conditioning but are resistant to subsequent extinction and display sustained activity; and “*extinction neurons*”, which are silent after fear conditioning but display CS-evoked activity after subsequent extinction. Neural populations that match the definitions of these three-types of neurons are not localized to specific regions; instead, they are redundantly distributed over the amygdala and mPFC: fear neurons have been found in the basal nuclei of the amygdala (BA) [27,28], lateral nuclei of the amygdala (LA) [28–31] and central nuclei of the amygdala (CEA) [32]; persistent neurons have been found in the BA [27,28] and LA [28,30,31]; and extinction neurons have been found in the BA [27,28], the group of intercalated cells (ITC) [32] and the vmPFC [33–35]. The following questions arise: How do these three neural populations interact? Furthermore, how do their interactions process both CS and uncertainly generated US inputs during partial reinforcement fear conditioning and generate an extinction-resistant fear response as output?

The vmPFC is widely considered to be a primary assembly of extinction neurons because it inhibits the amygdala through activating the GABAergic ITC [36,37]. In fact, activation of the vmPFC led to the suppression of CS-evoked fear memory [38,39]. Nevertheless, a recent optogenetic study showed that the vmPFC is necessary for the formation but not the expression of the extinction memory, suggesting that inhibitory sources other than the vmPFC could also suppress the fear memory [40]. Thus, the functional role of the vmPFC remains controversial.

Based on these neural findings, this study sought a possible explanation of the PREE by hypothesizing that a combination of fear, persistent and extinction neurons plays an important role in the PREE. To test this hypothesis, we first developed a mathematical model of a neural circuit based on three neural units with the basic properties of the fear, persistent and extinction neurons. We then presented how uncertainly generated US inputs were processed in the neural circuit model, with a particular eye to the PREE. Finally, an extension of the model provided a plausible explanation for the controversial role of the vmPFC in the formation of extinction memory.

## Models

To examine how fear memory is learned in fear conditioning with partial reinforcement and is resistant to extinction, we developed two neural circuit models. We first constructed a basic model of a neural circuit consisting of fear, persistent and extinction neurons, while each type of neurons redundantly distributed over amygdala and mPFC was not fully distinguished (Fig 1D). We then extended the basic model to include nuclei in the amygdala (LA, CEA and ITC) and vmPFC (Fig 1E).

### Basic model of the neural network with fear, persistent and extinction neurons

The model mainly consisted of fear and persistent neurons in the amygdala and extinction neurons that were considered to be within the vmPFC (Fig 1D). Note that extinction neurons were also found in the amygdala and that those neurons receive synaptic inputs from the vmPFC [28,37]. Here, we simply addressed populations of fear, persistent and extinction neurons as single representative units: fear, persistent and extinction neural units. Thus, the activity of each neural unit represents the averaged firing rate of each neural population. In the model, these neural units composed two kinds of networks for neural activity and learning signals.

In the neural activity-regulating network, all units were activated by excitatory synaptic input from the CS, and the extinction neural unit inhibited the fear neural unit (black line in Fig 1D). Behavioral fear responses were simply represented by the activity of the fear neural unit, reflecting the fact that the firing rate of fear neurons is well correlated with the freezing response of animals [41]. The activities of the fear neural unit (*F*), persistent neural unit (*P*), and extinction neural unit (*E*) at trial *t* were described by

$$F(t)=w_{F}(t)CS(t)-w_{F,E}E(t),$$ (1)

$$P(t)=w_{P}(t)CS(t),$$ (2)

$$E(t)=w_{E}(t)CS(t),$$ (3)

where *CS* denotes the CS input, which was 1 when the CS was provided and 0 otherwise; *w*_*F*,*E*_ denotes the synaptic weight with which the extinction neural unit inhibits the fear neural unit; and *w*_*F*_, *w*_*P*_ and *w*_*E*_ indicate the synaptic weights of the CS-related inputs (black lines in Fig 1D).

In the learning signal-regulating network, the learning signals inducing synaptic plasticity of *w*_*F*_, *w*_*P*_ and *w*_*M*_ were computed in a neural activity-dependent manner (blue line in Fig 1D). These weights were updated on a trial-by-trial basis after each CS-US presentation by the following synaptic plasticity rules:

ΔwF=αFCS(t)[US(t)−F(t)]+, (4)

ΔwP=αPCS(t)[US(t)−P(t)]+, (5)

ΔwE=αECS(t)[F(t){P(t)−US(t)−E(t)}]+, (6)

where *α*_*F*_, *α*_*P*_ and *α*_*E*_ denote the learning rates; *US* is the intensity of US input; and [*x*]_+_ is a rectified linear function: [*x*]_+_ is 0 and *x* when *x*<0 and *x*≧0, respectively. The brackets ([]_+_) in the equations represent the learning signals that regulate the gain of synaptic plasticity [42] (blue lines in Fig 1D).

In this scheme of synaptic plasticity, the activity of the fear, persistent and extinction neural units can be interpreted to represent ‘prediction of severity (threat)’, ‘prediction of US intensity’ and ‘prediction of safety (no presentation of US; we subsequently refer to this case as ‘no-US’)’, respectively. The fear neural unit receives two inputs (eq (1)): the CS input, which is a cue signal for the subsequent US, and the inhibitory input from the extinction neural unit, which predicts the degree of safety. This suggests that the fear neural unit predicts the net severity. In eq (4), the synaptic weight of the CS input to the fear neural unit, *w*_*F*_, is modulated, according to the Rescorla-Wagner learning rule [43,44], based on the prediction error of the net severity. The persistent neural unit responds to the sole CS input (eq (2)). The synaptic weight of the CS input to the persistent neural unit, *w*_*P*_, is also modified according to Rescorla-Wagner learning (eq (5)), which allows the persistent neural unit to predict the US intensity (*P* = *US*). To reflect that actual extinction neurons come to respond to the CS after extinction training [28], the extinction neural unit was assumed to encode safety (no-US) in our model. In eq (6), the synaptic weight of the CS input to the extinction neural unit, *w*_*E*_, is modified by the prediction error of the safety, where *P*−*US* represents the actual safety (no-US), *e*.*g*., after acquisition of fear memory, *P*−*US* = 0 when US, and *P*−*US* = learned US intensity when no-US.

**Fig 2:**
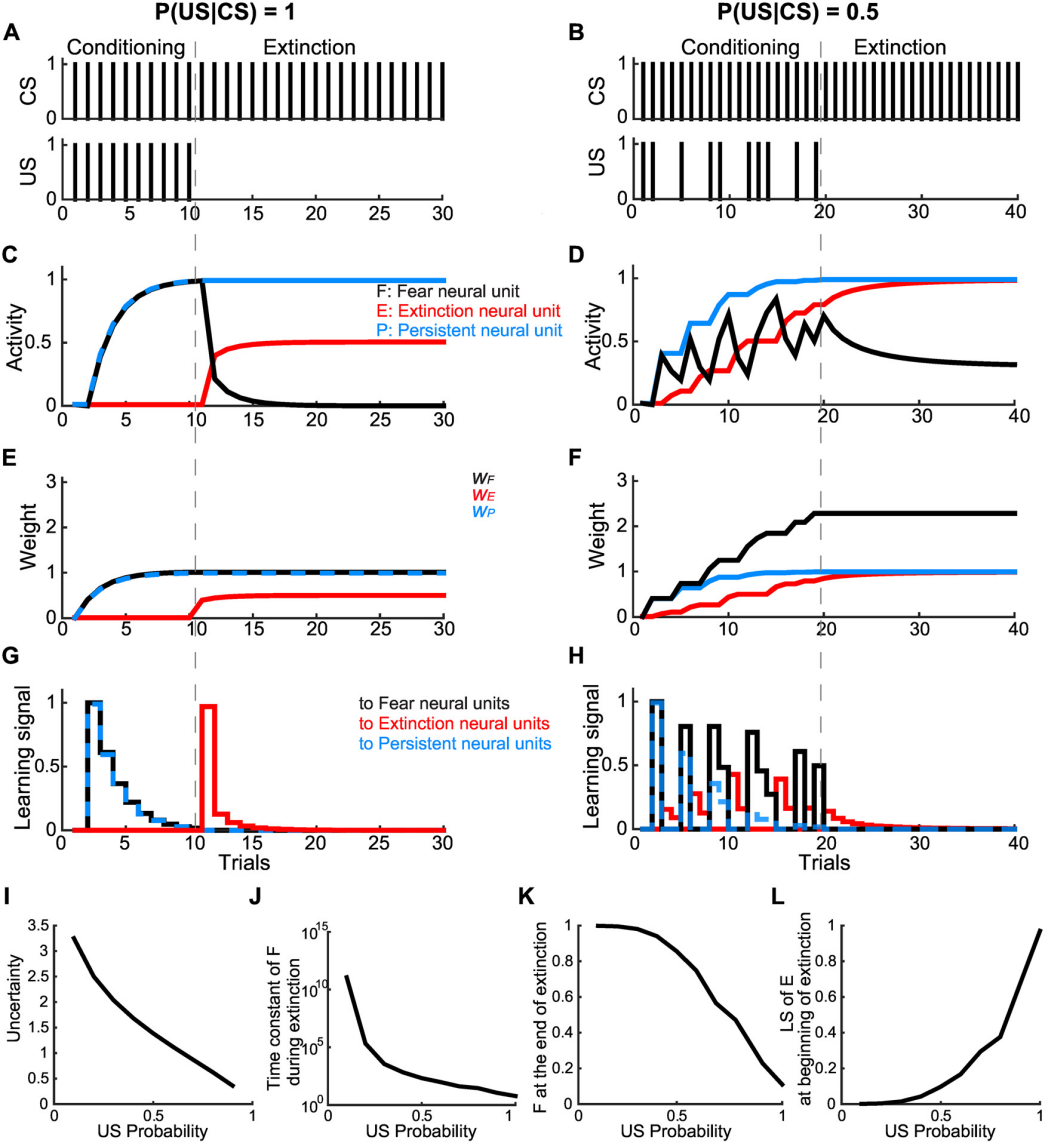
Fear memory acquired during the full and partial reinforcement schedules in the basic model. Basic model simulation results for the full and partial reinforcement schedules are presented in the left **(A, C, E and G)** and right **(B, D, F and H)** columns, respectively. **(A, B)** CS and US schedules during fear conditioning and extinction. **(C, D)** The black, blue and red lines indicate the activity of the fear, persistent and extinction neural units, respectively. **(E, F)** The black, blue and red lines indicate the synaptic weights of the CS-related inputs to the fear, persistent and extinction neural units, respectively. **(G, H)** The black, blue and red lines indicate the learning signals that changed the weights of CS-related synaptic inputs to the fear, persistent and extinction neural units, respectively. Note that overlapping lines were changed to dashed lines to make them visible. **(I-L)** The uncertainty of the next US observation **(I)**, the time constant of fear memory decline **(J)**, the relative fear-related neural activity at the conclusion of extinction **(K)** and the learning signal received by the extinction neural unit at the beginning of extinction **(L)** vary with the probability of the US. The time constant **(J)** was evaluated by fitting time course of the fear neural unit activity during extinction with *F = F*_*1*_exp(*-t/τ*)+*F*_*0*_, where *F*_*0*_ and *F*_*1*_ indicate positive constants, and *t* and *τ* indicate the number of extinction trials and the time constant, respectively.

In the model, the CS-US pair was applied in a sequential training manner during fear conditioning and extinction. During fear conditioning, the CS and US were repeatedly paired in the full reinforcement case (*US* = 1) (Fig 2A), whereas the US was probabilistically paired with the CS in the partial reinforcement case (P(*US* = 1|*CS =* 1)<1) (Fig 2B). During extinction, the CS was repeatedly applied without being paired with the US (*US* = 0).

### Extended neural network model including the amygdala and vmPFC

The basic model was extended by additionally introducing two factors: another extinction neural unit and multiple timescales of synaptic plasticity. These two factors were implied by a recent optogenetic study [40]. Silencing of the vmPFC (i.e., IL in rodents) had no effect on CS-evoked behavioral responses during extinction, suggesting another inhibitory source of fear memory other than the vmPFC. In addition, silencing of the vmPFC during extinction impaired the retrieval of extinction memory, suggesting that formation and consolidation of extinction memory are regulated by fast and slow timescales of synaptic plasticity.

The extended model took into account a heterogeneous collection of subregions in the amygdala as well as in the vmPFC [45], in which the LA and CEA were represented as the persistent and fear neural units, respectively, whereas the ITC and the vmPFC were represented as extinction neural units (Fig 1E). All subregions receive CS-related synaptic inputs through the thalamus and cortex. The ITC received CS-related input and input from the vmPFC, and it inhibited the CEA. Behavioral fear response was represented by the activity of the CEA. The activity of the CEA (*F*), LA (*P*), vmPFC (*E1*) and ITC (*E2*) are described by

$$F(t)=w_{F}(t)CS(t)-w_{F,E2}E2(t),$$ (7)

$$P(t)=w_{P}(t)CS(t),$$ (8)

$$E1(t)=w_{E1}(t)CS(t),$$ (9)

$$E2(t)=w_{E2}(t)CS(t)+w_{E2,E1}E1(t),$$ (10)

where *w*_*i*_ (*i =* {*F*, *P*, *E1*, *E2*}) indicates the activity-dependent modifiable synaptic weight of CS-related input to *i* (black lines in Fig 1E) and *w*_*i*, *j*_ indicates the constant synaptic weight of input from *j* to *i*. The synaptic plasticity rules were the same as those in the basic model (eqs (4–6)), except that two different timescale dynamics were introduced to *w*_*E2*_ to represent early- and late-phase plasticity [46,47]:

ΔwF=αFCS(t)[US(t)−F(t)]+, (11)

ΔwP=αPCS(t)[US(t)−P(t)]+, (12)

ΔwE1=αE1CS(t)[F(t){P(t)−US(t)−E1(t)}]+, (13)

ΔwE2=αE2CS(t)[F(t){P(t)−US(t)−E2(t)}]+−βE2(wE2(t)−wE2∞(t)), (14)

where *α*_*i*_ (*i =* {*F*, *P*, *E1*, *E2*}) denotes the learning rate, and *β*_*E2*_ denotes the relaxation rate, of *w*_*E2*_(*t*) to *w*_*E2*_^∞^(*t*). The first terms correspond to early-phase plasticity, which depend on the learning signals (blue lines in Fig 1E) indicated by brackets ([]_+_). The second term in eq (14) represents late-phase plasticity, where *w*_*E2*_^∞^ indicates the weight capacity, the dynamics of which are described by

ΔwE2∞=αE2∞CS(t)E1(t)−βE2∞(wE2∞(t)−wE2(t)), (15)

where *α*_*E2*_^∞^ and *β*_*E2*_^∞^ indicate the learning rate and relaxation rate, respectively. Here, the learning signal to *w*_*E2*_^∞^ is *E1*(*t*). According to eqs (14) and (15), *w*_*E2*_ is consolidated to *w*_*E2*_^∞^, and *w*_*E2*_^∞^ is also relaxed to *w*_*E2*_ depending on the activity of the vmPFC, *E1*(*t*).

What are the molecular substrates of early- and late-phase plasticity? Early-phase long-term potentiation (LTP) is regulated by Ca^2+^ signaling-regulated phosphorylation of AMPA-R on endosomes, which induces the exocytosis and membrane accumulation of AMPA-R [48]. In contrast, late-phase LTP is thought to be regulated by gene expression with slow dynamics, in which proteins are newly synthesized in the soma, actively transported to spines and inserted into the postsynaptic density (PSD) [49,50]. Thus, *w*_*E2*_ and *w*_*E2*_^*∞*^ in eqs (14 and 15) correspond to the total number of AMPA-Rs on membrane and the size of PSD, *i*.*e*., AMPA-R capacity, respectively. Then, each term in eqs (14 and 15) can be interpreted as the following biological processes: The first term in eq (14) represents the early-phase LTP, *i*.*e*., an increase in the total AMPA-Rs, regulated by the learning signal. The first term in eq (15) represents an increase in the AMPA-R capacity, regulated by the vmPFC. The second term in eq (14) represents spontaneous cycling (*i*.*e*., exocytosis and endocytosis) of the total AMPA-Rs, converging to the AMPA-R capacity. The second term in eq (15) represents spontaneous cycling of the AMPA-R capacity (*i*.*e*., synthesis and degradation of proteins in the PSD) depending on the total AMPA-Rs.

## Results

### Basic neural circuit model with fear, persistent and extinction neurons

To determine the basic dynamics of the fear, persistent and extinction neurons, we started with the basic model (Fig 1D). In the simulation of fear conditioning with full reinforcement and subsequent extinction (Fig 2A), the CS-evoked activities of the fear, persistent and extinction neural units were consistent with the respective properties of fear, persistent and extinction neurons in the amygdala (Fig 2C) [26,28,32]. In addition, the activity of the fear neural unit well represented the behavioral freezing rate as observed in fear conditioning and extinction [28]. Thus, the basic model reproduced the behaviors of the fear, persistent and extinction neurons.

Next, we performed simulations in the partial reinforcement case (Fig 2B). During the fear conditioning with partial pairing of the US, the activity of the fear neural unit increased when the US was presented and decreased when it was not (no-US), but the overall activity tended to increase; in contrast, the activities of the persistent and extinction neural units increased only when US and no-US were presented, respectively (Fig 2D). During the subsequent extinction phase, we observed the PREE (Fig 2D): the activity of the fear neural unit slowly decreased with residual activity, in contrast to what was observed in the full reinforcement case (Fig 2C). Consistently, residual neural firing has been observed in the amygdala after the extinction training of partially reinforced fear memory [26]. We also found that the synaptic weight from the extinction neural unit to the fear neural unit, *w*_*F*,*E*_, was critical for the PREE (S1 Fig).

In addition, we found that the PREE-like effect could be observed during successive full reinforcement conditioning and extinction, being equivalent to partial reinforcement conditioning overall (S2 Fig). As conditioning and extinction repeat, the residual activity of the fear neural unit accumulates and becomes saturated. In fact, it has been seen in the literature that the re-conditioned fear memory exhibited substantial resistance to re-extinction [51–54]. Hence, the basic model based on crosstalk between the fear, persistent and extinction neurons processed the probabilistic nature of the pairing, *i*.*e*., the uncertainty.

What causes the difference in the extinction of fully and partially reinforced fear memories? After fear conditioning with full reinforcement, CS-evoked activity of the persistent neural unit converged to the US intensity, *i*.*e*., *P* = 1, whereas the extinction neural unit showed no activity, *i*.*e*., *E* = 0 (Fig 2C). Thus, the no-US at the beginning of the extinction, *i*.*e*., *US* = 0, led to the maximum level of the learning signal to the extinction neural unit (red line in Fig 2G), which was proportional to *P*-*US*-*E*, resulting in the extinction and fear neural units showing a rapid increase and decrease in their respective CS-evoked activities. After fear conditioning with partial reinforcement, *P* = 1, the same as after fear conditioning with full reinforcement, whereas the extinction neural unit showed a certain level of activity, *i*.*e*., *E*≧0 (Fig 2D). Thus, at the beginning of the extinction, *i*.*e*., *US* = 0, the learning signal to the extinction neural unit, which was proportional to *P*-*US*-*E*, exhibited a lower level than that after fear conditioning with full reinforcement (red line in Fig 2H). Therefore, the extinction neural unit could not produce enough of an increase in the CS-evoked activity to inhibit the fear neural unit. This is a scenario of the PREE.

In addition, we found that the learning signal to the extinction neural unit was correlated with the degree of ‘surprise’ from a statistical standpoint; after fear conditioning with full reinforcement, the no-US input at the beginning of the extinction phase was unpredictable, leading to a relatively large degree of surprise (red line in Fig 2G). In contrast, after fear conditioning with partial reinforcement, the no-US input at the beginning of the extinction phase was predictable, leading to a relatively small degree of surprise (red line in Fig 2H).

We also quantitatively evaluated the degree of surprise by developing a statistical inference model based on sequential updating of Bayesian logistic regression (see S1 Text). Then, we found that the learning signal to the extinction neural unit was positively correlated with the degree of surprise (see S3 and S4 Figs).

We further investigated the effect of uncertainty during fear conditioning on the PREE. The uncertainty was evaluated in terms of the Shannon entropy of the probability distribution for the waiting time (number of trials: *n*∈{1, 2, …}) until the next US observation, *P*(*n*) = *P*(*US* = 1|*CS* = 1)(1−*P*(*US* = 1|*CS* = 1))^*n*−1^ [55]. Obviously, the uncertainty monotonically increased as *P*(*US* = 1|*CS* = 1) decreased (Fig 2I). With a high degree of uncertainty (low P(*US* = 1|*CS* = 1)), the fear neural unit was highly resistant to extinction with a longer time constant (Fig 2J), and its residual activity at the end of the extinction phase remained high (Fig 2K), indicating that uncertainty facilitated the PREE. This is because the activity of the extinction neural unit was almost saturated after fear conditioning and did not increase enough to inhibit the fear neural unit during the extinction phase (Fig 2D) due to the weakness of the learning signal to the extinction neural unit (Fig 2L).

### Extended neural circuit model with the amygdala and vmPFC

In our basic model, the extinction neural unit, which presumably corresponds to the vmPFC, was the unique source of inhibition of the fear memory, indicating that the extinction neural unit was required for both the formation and retrieval of the extinction memory. Consistently, it has been shown that optogenetic activation of the vmPFC (i.e., IL in rodents) reduces the fear response; in particular, vmPFC activation during the extinction phase facilitated the consolidation of the extinction memory [40]. However, vmPFC silencing experiments did not produce data consistent with the idea that the vmPFC is the unique source of inhibition of the fear memory; optogenetic silencing of the vmPFC during extinction impaired the retrieval of the extinction memory the next day, whereas silencing the vmPFC during retrieval had no effect, indicating that the vmPFC is necessary for the formation of the extinction memory but not for its retrieval [40]. This hypothesis has also been supported by a vmPFC lesion study [56]. Moreover, silencing the vmPFC during the extinction phase did not change the CS-evoked behavioral responses compared with those in the normal condition, although it impaired the retrieval of the extinction memory the next day, suggesting that formation and consolidation of the extinction memory are regulated by fast and slow timescales of synaptic plasticity.

**Fig 3:**
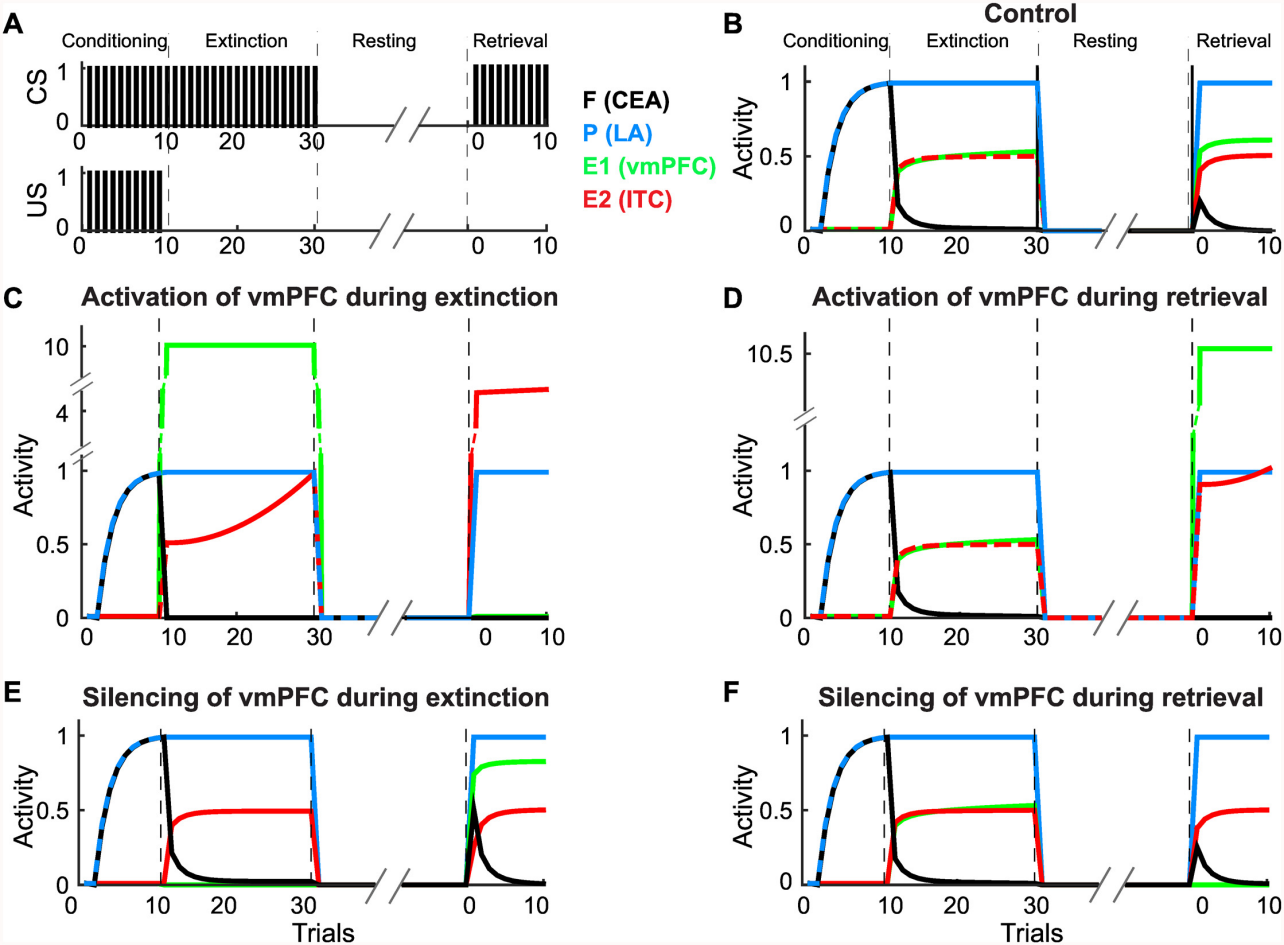
Fear memory in the extended model when the vmPFC was activated and silenced. **(A)** Schedules of CS and US; the presentation schedules for the CS and US during fear conditioning and extinction were the same as those used in the basic model (Fig 2A), but the resting phase, during which neither the CS nor the US was presented, and the retrieval phase, during which only the CS was presented to evaluate extinction memory, were set after extinction. **(B-F)** Extended model simulation results for the control condition **(B)**, vmPFC activation during extinction **(C)**, vmPFC activation during retrieval **(D)**, vmPFC silencing during extinction **(E)** and vmPFC silencing during retrieval **(F)**. The blue, green, red and black lines indicate the activity of the LA (persistent neurons), vmPFC (extinction neurons), ITC (another group of extinction neurons) and CEA (fear neurons), respectively. Note that overlapping lines were changed to dashed lines to make them visible.

Here, we aimed to reproduce the new findings of this optogenetic study by extending the basic model. In the extended model, we considered a neural circuit consisting of nuclei in the amygdala and mPFC; in this model, the LA, CEA and vmPFC were simply represented by the persistent, fear, and extinction neural units, respectively. The extended model also included the ITC as another extinction neural unit (Fig 1E) (see Model). The extended model could provide minimal understanding of the amygdala-mPFC neural circuit, although a simple correspondence between brain regions and functions, *e*.*g*., the CEA as fear neurons, the LA as persistent neurons and both the ITC and vmPFC as extinction neurons, could be an oversimplification because fear, persistent and extinction neurons are distributed throughout the amygdala and mPFC. In this model, we also developed a synaptic plasticity mechanism for early-phase memory formation and late-phase memory consolidation (see Model). In the simulation, after the schedule of CS-US pairings used in the basic model was repeated, no-CS and no-US resting trials as well as subsequent retrieval trials with only the CS were performed (Fig 3A).

This extended model showed essential behaviors of fear memory acquired through fear conditioning with full reinforcement and extinction (Fig 3B). At the retrieval of the extinction memory after the resting phase, spontaneous recovery of the fear memory occurred to a small extent, as commonly observed after long intervals [57–59]. This is because during the resting phase, the synaptic weight to ITC, *w*_*E2*_, settled down to the weight capacity, *w*_*E2*_^*∞*^, due to the slow dynamics of the late-phase LTP (S8B Fig). We also confirmed that the extended model generated the PREE in partial reinforcement conditioning (S5 Fig) and the PREE-like effect in successive full reinforcement conditioning and extinction (S6 Fig), consistent with the basic model (Fig 2D and S2 Fig).

The extended model consistently reproduced the experimental results (see Figs 2B, 2C, 3C, and 4B in Do-Monte et al. [40]) observed for the activation and silencing of the vmPFC during extinction and retrieval (Fig 3C–3F). Activation of the vmPFC reduced the expression of fear during both extinction (Fig 3C) and retrieval (Fig 3D), and the vmPFC activation during extinction also facilitated the subsequent consolidation of the extinction memory during the resting phase, causing no recovery of the fear memory at the retrieval (Fig 3C). When the vmPFC was suppressed during extinction, even though there was no effect on the extinction of the fear memory, the extinction memory was not consolidated during the resting phase, leading to significant spontaneous recovery of the fear memory (Fig 3E). This finding is consistent with recent reports [40,56] and suggests that extinction learning is regulated by separate synaptic plasticity mechanisms consisting of early-phase memory formation that is independent of the vmPFC and late-phase memory consolidation that depends on the vmPFC, as assumed in our model. In addition, consistent with recent reports [40], suppressing the vmPFC during retrieval did not affect the extinction memory (Fig 3F). Taken together, the consistency between previous experimental reports and our simulation supports the validity of our extended model, which included another inhibitory source of fear memory in addition to the vmPFC as well as separate synaptic plasticity mechanisms underlying early-phase memory formation independent of the vmPFC and late-phase memory consolidation dependent on the vmPFC.

### Model prediction: Procedure to diminish extinction-resistant fear response

The partially reinforced fear memory could not be fully inhibited by the extinction training (Fig 2D and S5D Fig), which is reminiscent of exposure therapy-resistant anxiety disorder, panic disorder and post-traumatic stress disorder (PTSD) [60]. Here, we explored a new procedure to relieve the resistance to extinction, and we then tested it based on our model. Diminishing the extinction-resistant fear response would require a considerable increase in the activity of the extinction neural unit, which is driven by the learning signal, as described by eq (6). According to this equation, increasing the learning signal requires an increase in the activity of the fear and persistent neural units. Thus, this observation suggested a ‘shock procedure’ in which the CS was paired with a stronger US; this pairing was applied before further extinction training.

**Fig 4:**
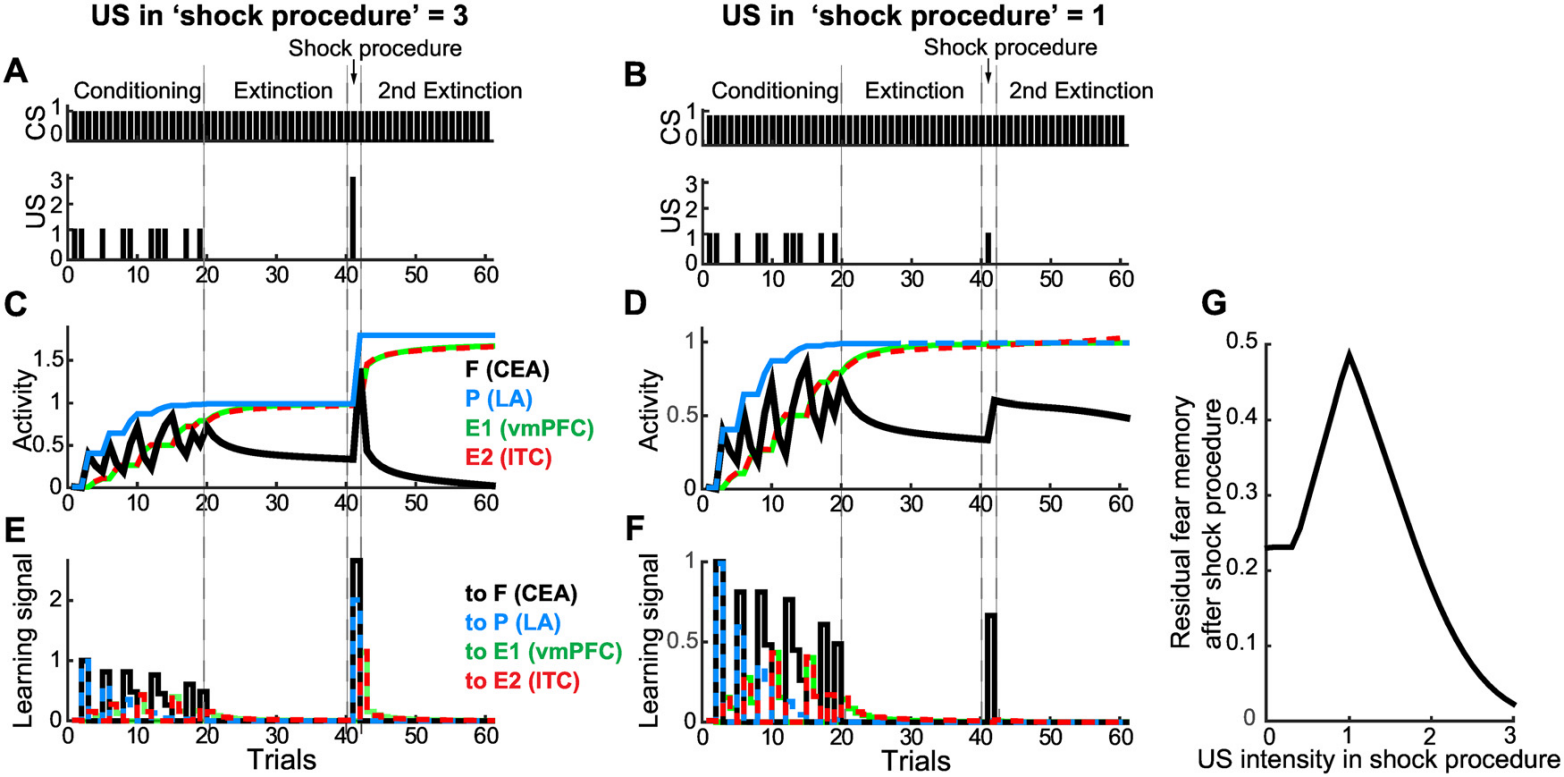
Shock procedure in the extended model. **(A, B)** CS and US schedules; after extinction training of the partially reinforced fear memory, an additional CS-US pairing was applied, with the US being three times stronger **(A)** or the same intensity **(B)**. **(C, D)** The blue, green, red and black lines indicate the activities of the LA (persistent neurons), vmPFC (extinction neurons), ITC (another group of extinction neurons) and CEA (fear neurons), respectively. **(E, F)** The blue, green, red and black lines indicate the learning signals that changed the weights of CS-related synaptic input to the LA, vmPFC, ITC and CEA, respectively. Note that overlapped lines were changed to dashed lines to make them visible. **(G)** Final activity levels of the CEA (fear neural unit) after re-extinction training were plotted depending on the US intensity.

We then tested this shock procedure by using the extended model. The activity of the fear neural unit was first rapidly elevated due to high intensity of the US (Fig 4A) and then rapidly decreased to almost 0 during the subsequent extinction (black line in Fig 4C), indicating that the extinction-resistant fear memory was completely inhibited. When the shock procedure employing an US of the same intensity was applied (Fig 4B), the fear memory was conversely reinforced (Fig 4D), suggesting that the intensity of the US is a critical determinant for the effectiveness of the shock procedure. The differences in the outcomes of these cases were due to different levels of learning signals to two extinction neural units (E1 and E2) at the beginning of the second extinction (green and red lines in Fig 4E and 4F). We evaluated the effectiveness of the shock procedure for various US intensities by final activity level of CEA (the fear neural unit) after re-extinction training (Fig 4G). We confirmed that the shock procedure was also successful in the basic model (S7 Fig).

## Discussion

We presented two neural circuit models based on the recent discovery of three distinct types of neural populations (fear, persistent and extinction neurons) to reveal how uncertainty is processed in the amygdala-mPFC circuit, with a particular interest in the PREE. In our models, for the sake of simplicity, we addressed three types of neurons (fear, persistent and extinction neurons) distributed over subdivisions of the amygdala and mPFC. The in vivo neural circuit must be more complicated than what we assumed in our models. However, the minimalist model we developed was very informative and provided a system-level understanding of the amygdala-mPFC circuit in fear conditioning and extinction. That model provided the insight that the degree of surprise in an uncertain situation is encoded through the combined activity of the fear, persistent and extinction neurons as well as an experimentally testable prediction regarding how extinction-resistant fear memory could be diminished.

### Extinction as inhibitory learning

It has been generally accepted that extinction is a form of inhibitory learning, which is opposite from an erasure or forgetting of fear memory [61]. In fact, extinguished fear responses recover under various circumstances. For example, an extinguished fear response spontaneously recovers after a long time, *e*.*g*., several days [58], and also reappears after exposure to the US without the CS, known as reinstatement [62]. It has been reported that extinction training inhibited the fear responses but could not erase the fear memory in adult rat, although erasure of fear memory may occur during early stages of postnatal development [63,64]. Consistently, conditioned fear memory in our model was not erased by a decrease in synaptic weights but was inhibited by extinction neurons.

### Encoding

Recent physiological studies have identified neural populations with distinct firing characteristics, such as fear, persistent and extinction neurons, in the amygdala [27–32]. These findings suggest that information processing in the amygdala may take place through these neural populations. However, the computational role of each neural population in fear conditioning and extinction has not been well studied. In this study, we presented the neural implementation based on these neural populations and proposed the types of information that are encoded and processed through interactions between these neural populations: the fear, persistent, and extinction neurons encode the prediction of net severity, of US intensity and of safety (no-US), respectively, and the weights of their synaptic inputs are modulated by the corresponding prediction errors.

Consistent with the persistent neurons in our model, a previous report showed that CS-evoked activity of the persistent neurons in the LA does not further increase after reconditioning, suggesting that persistent neurons represent the memory of the US intensity [30]. Consistent with the extinction neuron in our model, a human fMRI study showed that the vmPFC uniquely encodes safety accompanied by the CS during extinction [24], suggesting that the CS synaptic input to the vmPFC could be plastically regulated by ‘prediction error of safety’. This proposed encoding mechanism could be further validated by experiments, such as electrophysiological recording of neural firing or the labeling of cFos immunoreactivity during partial reinforcement fear conditioning and extinction.

### Learning signals & neuromodulators

It can be speculated that the learning signals in our model could be implemented through neuromodulators, *e*.*g*., dopamine, serotonin, noradrenaline, acetylcholine, norepinephrine and oxytocin [42]. In general, the release of neuromodulators is associated with particular mental states, *e*.*g*., reward, positive and negative emotions, happiness, motivation, attention and arousal [65]. Neuromodulators regulate neuronal firing and the efficacy of synaptic plasticity [66]. In particular, dopamine has been extensively investigated, and it is widely accepted that dopamine release from the ventral tegmental area (VTA) is a specific response to reward-related prediction error, *i*.*e*., the acquisition of a greater reward than expected [67,68] and that the dopamine release facilitates the synaptic plasticity that underlies the association between sensory input (the CS) and reward (the US) [69–72]. Based on these facts, reinforcement learning theory has suggested that animals perform temporal difference (TD) learning [73–75] because the basal ganglia, which is involved in decision making, receives dense axonal projections from the VTA and exhibits dopamine-dependent plasticity of synaptic inputs from the cortex [71]. However, it has been known that dopaminergic neurons show firing responses not only to rewards but also to aversive stimuli [76] and, moreover, show diverse firing patterns that may encode prediction errors of other valences [77,78]. In addition, the VTA was recently suggested to be composed of anatomically and functionally heterogeneous dopaminergic neurons whose axons project to different regions, including the amygdala and mPFC [79]. Taken together, dopamine signals to different neural populations may represent different meanings, such as the prediction error of net severity, of US intensity and of safety, as assumed in our model.

### Anxiety disorders

Fear conditioning has been used as a model system for anxiety disorders such as panic disorder, PTSD and obsessive-compulsive disorder (OCD) [80]. Traditional exposure therapy, which corresponds to extinction training, is an effective cure for anxiety disorders in some patients [81], but some severe patients also show strong resistance to exposure therapy [82,83]. Moreover, anxiety disorders may be worsened by occasionally experienced negative social reactions [84,85], which are akin to partial reinforcement experiences, and become strongly resistant to exposure therapy, similar to the PREE. Thus, we think that the widely used fear conditioning with full reinforcement, in which the acquired fear memory can be easily diminished by extinction training, is rare in real life and thus is not a good model for understanding anxiety disorders; instead, fear conditioning with partial reinforcement, which results in an extinction-resistant fear memory, is a more realistic model for neuroscience research on anxiety disorders and should improve the translatability of results [18,26].

To relieve extinction-resistant fear memory, we proposed a ‘shock procedure’ based on our model. The fear memory was diminished by extinction if a stronger US was paired with the CS before the extinction procedure (Fig 4 and S7 Fig). In the shock procedure, although the fear memory is temporarily strengthened by the stronger US, the subsequent extinction training becomes effective, suggesting that an increase in activity in the amygdala (persistent and fear neurons) or in the learning signal to the vmPFC is key for effective extinction training. The shock procedure can be tested in animal experiments, but employing a stronger US as part of the shock procedure may raise ethical concerns for humans. It can be intuitively interpreted that the animal cannot comprehend the rule change to extinction after fear conditioning with partial reinforcement, whereas after the shock procedure with a strong US, in contrast, the animal can internalize the pronounced rule change to extinction, thus allowing the fear memory to be extinguished. The proposed ‘shock procedure’ may provide insight not only for the development of new therapies but also for understanding the neural mechanisms of fear memory extinction.

### Previous theoretical models

Classical conditioning has been computationally modeled in a number of ways. In the field of behavioral psychology, ‘former learning theory’, which defines mathematical embodiments to describe learning and behavioral phenomena, has been tested [86]. The Rescorla-Wagner model was a seminal former learning theory that described an association between CS and US controlled by prediction error as a learning signal [43]. Since then, many alternative models have been proposed to reproduce many observed phenomena in classical conditioning and extinction [87–91]. However, these models failed to explain the PREE. Moreover, these models did not fully describe their neural mechanisms, although several models can be implemented using neural networks [92,93].

‘Reinforcement learning’ was proposed as an extension of the Rescorla-Wagner model; in this system, which animals explore optimal behavioral strategies by interacting with their environment to maximize the accumulated reward over time [94]. Sutton and Barto proposed temporal difference (TD) learning, in which the prediction of expected cumulative future reward was updated by its prediction error, called TD error [94]. The framework of TD learning reproduced classical conditioning and extinction [75] but not the PREE. To account for the PREE, TD learning was extended by two models [9,10]. Redish et al. [9] introduced a categorization process for inexperienced observations into new latent states, whereas Song et al. [10] introduced arousal signal-dependent learning to the existing TD learning model [95]. Although these TD learning-based models were successful in reproducing the PREE, how neural computation is performed by fear, persistent and extinction neurons has remained unclear.

Another aspect of computational modeling is ‘statistical decision theory’ [96]. The PREE has been addressed by Bayesian estimation of the US probability per trial or unit time [12,97,98]. This framework was extended to introduce latent causes [8]. Related to latent causes, Gershman et al. [99] developed a Bayesian inference model based on a categorization process of contexts [9]. This model was further extended by introducing a hidden Markov model (HMM), in which a particular context tended to persist over time, and this model successfully generated the PREE [11]. Although these models provided important concepts in light of statistical decision making, they did not describe the underlying neural mechanisms.

There have also been two types of approaches to computational models of neural circuits consisting of the amygdala and other brain regions. One approach is the firing-rate model, in which neural units represent the average firing rate of neurons, neural populations or brain regions [100–102]. Balkenius et al. [102] first developed a mathematical model in which fear memory was extinguished by inhibition of amygdalar activity by the orbitofrontal cortex, which is subdivision of the vmPFC, but their model did not focus on and hence failed to reproduce the PREE. Moustafa et al. [101] developed a neural circuit model consisting of the BLA, CEA, ITC and vmPFC (*i*.*e*., IL), combined with TD learning, in which synaptic plasticity was regulated by the TD error as a learning signal. In their study, however, the PREE was not explicitly addressed, though it was mentioned that their model exhibited the PREE only when extensive training trials were performed in the acquisition phase, with no detailed results.

The other approach is based on a spiking-neuron model, in which action potentials are simulated based on membrane voltage dynamics. Nair’s group has extensively developed biophysically realistic conductance-based models that express several types of firing patters that have been experimentally observed [103–106]. These studies addressed fear conditioning only with full reinforcement, not with partial reinforcement, while investigating the roles of synaptic input from the vmPFC to the ITC [106], interaction between prelimbic cortex (PC) in the mPFC and the BA [104], and synaptic inputs from thalamus and cortex to the LA [105]. On the other hand, Vlachos et al. [107] first proposed a large-scale neural network model of the BA by introducing populations of fear, persistent and extinction neurons, but that work did not address the PREE.

Compared with these previous models, our model is the first neural network model of the amygdala/vmPFC circuit that could satisfactorily explain the PREE, and it is based on the three types of neurons (fear, persistent and extinction neurons).

### Limitations and further extension

Finally, limitations of our model are worth mentioning. Recently, it has been reported that gradually reducing the frequency of the US (called gradual extinction) prevents the spontaneous recovery of fear memory when compared with full reinforcement of the US [108]. This fact suggested that not only frequency but also the temporal pattern of the US affects consolidation of extinction memory. However, our model cannot demonstrate the effect of gradual extinction on the spontaneous recovery of fear memory. This effect would be addressed by modeling leaky integration of learning signals, which we leave to our future work.

Although the mPFC is divided into several subregions including the dorsal mPFC (dmPFC) and vmPFC, our model only addressed the vmPFC. In contrast to the vmPFC, the dmPFC plays an important role in the acquisition of fear memory [16,109]. It has been reported that sustained activity of the dmPFC is correlated with extinction failure, which should be related to resistance to extinction [110]. Moreover, a recent electrophysiological study of monkeys investigated activity in the amygdala and dorsal anterior cingulate cortex (dACC), namely, the dmPFC in primates, during partial reinforcement fear conditioning and showed that correlated amygdala-dACC activity during fear conditioning determines resistance to extinction [26]. Further extension of the current model, possibly introducing additional fear neural units corresponding to the dmPFC, will be required.

This study addressed only cued conditioning, not contextual conditioning, in which context information is provided to the BA through the hippocampus [111,112]. In the BA, fear and persistent neurons are unidirectionally innervated from and reciprocally interact with the ventral hippocampus, respectively, whereas extinction neurons have no connectivity with the hippocampus [28]. In addition, BA fear neurons activate excitatory neurons in the dmPFC, whereas the ventral hippocampus inhibits the dmPFC via innervating inhibitory neurons in the dmPFC [113]. These facts suggest that three types of neurons in different nuclei play differential roles in integrating cue and context information. Modeling such differential roles is also left to our future work.

## Supporting Information

S1 Text A statistical inference model. (PDF)

S1 Fig In the basic model, the PREE depends on the synaptic weight from extinction to fear neural units. **(A)** Fear neural unit activity during the partial reinforcement fear conditioning (P(US|CS) = 0.5) and subsequent extinction was shown with various changes in *w*_*F*,*E*_. **(B)** Fear neural unit activity during the full reinforcement fear conditioning (P(US|CS) = 1) and subsequent extinction was shown with various changes in *w*_*F*,*E*_. Note that *α*_*E*_ was also concurrently changed such that *w*_*F*,*E*_ *α*_*E*_ = const. **(C)** Red and blue lines indicate the time constant of extinction after the full and partial reinforcement fear conditioning, respectively, varying *w*_*F*,*E*_. The time constant of extinction is defined in the legend of Fig 2. **(D)** Red and blue lines indicate the residual activity of the fear neural unit after the extinction following the full and partial reinforcement fear conditioning, respectively, varying *w*_*F*,*E*_.
(TIF)

S2 Fig Repeated alternations of conditioning and extinction induce PREE in the basic model. **(A)** The fear neural unit activity during successive conditioning and extinction with changes in *w*_*F*,*E*_. Note that *α*_*E*_ was concurrently changed such that *w*_*F*,*E*_ *α*_*E*_ = const. **(B)** The residual fear memory after each extinction session was plotted with the change in *w*_*F*,*E*_.
(TIF)

S3 Fig Fear memory as statistical inference. Simulation results for the statistical inference model with full and partial reinforcement schedules are presented in the left (A, C, and E) and right (B, D, and F) columns, respectively. (**A, B)** The CS and US events were applied according to the same schedule shown in Fig 2A and 2B. **(C, D)** The black lines indicate the US probability estimated by logistic regression with sequential Bayesian updating. **(E, F)** The blue and red lines indicate the degree of surprise for the US and no-US, which is measured as the amount of information and calculated as −log*P*(*US*) and −log(1−*P*(*US*)), respectively.
(TIF)

S4 Fig Comparison between the basic neural circuit model and the statistical inference model. **(A)** The black line indicates the uncertainty of the next US observation as a function of the probability of the US. **(B)** The black and blue lines indicate the time constant of fear memory decline during extinction as a function of the probability that the US will be presented during fear conditioning in the basic neural circuit model and the statistical inference model, respectively. **(C)** The black and blue lines indicate the fear memory at the end of extinction as a function of US probability during fear conditioning in the basic neural circuit model and the statistical inference model, respectively. **(D)** The black and blue lines indicate the surprise associated with the no-US, *i*.*e*., the learning signal to the extinction neural unit in the basic neural circuit model and the amount of information associated with a no-US observation in the statistical inference model, respectively, as a function of US probability during fear conditioning. **(E, F)** Comparison between learning signals in the basic neural circuit model and surprise in the statistical inference model when the same US schedule was applied in both models. Each dot in **(E)** represents the relationship between ‘learning signals to CS-related synaptic inputs to fear and persistent neural units’ and ‘surprise for US’ during fear conditioning (blue dots) at each trial, and each dot in **(F)** represents the relationship between ‘learning signals to CS-related synaptic inputs to the extinction neural unit’ and ‘surprise for no-US’ during fear conditioning (red dots) and extinction (magenta dots) at each trial. Note that during extinction, the surprise associated with the US and the learning signal to the fear neural unit are both 0 due to the absence of US input. Therefore, this relationship is not included in **(E)**.
(TIF)

S5 Fig Fear memory for the full and partial reinforcement schedules in the extended model. Simulation results for the extended model with full and partial reinforcement schedules are presented in the left (**A, C and E**) and right (**B, D and F**) columns, respectively. **(A, B)** CS and US schedules during fear conditioning and extinction. **(C, D)** The blue, green, red and black lines represent the activity of the LA (persistent neurons), vmPFC (extinction neurons), ITC (another group of extinction neurons) and CEA (fear neurons), respectively. Note that the green lines are almost invisible because they overlap with the red lines. **(E, F)** The blue, green, red and black lines represent the learning signals that change the weights of CS-related synaptic inputs to the LA, vmPFC, ITC and CEA, respectively. Note that the blue and green lines are almost invisible because they overlap with the black and red lines, respectively. Note that each panel looks the same as Fig 2A–2D, 2G and 2H, which correspond to the basic model, because the same values were used for the parameters (S1 Table).
(TIF)

S6 Fig Repeated alternations of conditioning and extinction induce the PREE in the extended model. **(A)** The fear neural unit activity during successive conditioning and extinction with changes in *w*_*F*,*E2*_. Note that *α*_*E2*_ was concurrently changed such that *w*_*F*,*E2*_ *α*_*E2*_ = const. **(B)** The residual fear memory after each extinction session was plotted against the change in *w*_*F*,*E2*_.
(TIF)

S7 Fig Shock procedure in the basic model. **(A, B)** CS and US schedules; after the extinction training for the partially reinforced fear memory, an additional CS-US pairing was applied, in which the US was three times stronger (**A)** or the same intensity **(B)**. (**C, D)** The black, blue and red lines represent the activity of the fear, persistent and extinction neural units, respectively. **(E, F)** The black, blue and red lines represent the learning signals to the fear, persistent and extinction neural units, respectively. **(G)** Effect of US intensity on the effectiveness of the shock procedure. Note that each panel looks the same as those in Fig 4, which corresponds to the extended model, because the same values were used for the parameters (S1 Table).
(TIF)

S8 Fig Activation and silencing of vmPFC using the extended model. **(A)** The same as Fig 3A. **(B-F)** Changes in the synaptic weights in Fig 3. The blue, green, red, and black lines represent the early-phase plasticity-regulated weight of CS-related synapses to the LA (persistent neurons), vmPFC (extinction neurons), ITC (another group of extinction neurons) and CEA (fear neurons), respectively, and the grey lines represent the late-phase plasticity-regulated weight of CS-related synapses to the ITC.
(TIF)

S1 Table Parameters used in the basic and extended models. (TIF)
