# Supplementary material for: Uncertainty-Dependent Extinction of Fear Memory in an Amygdala-mPFC Neural Circuit Model
Source: PLoS Comput Biol. 2016 Sep 12;12(9):e1005099. doi: 10.1371/journal.pcbi.1005099 (PMC5019407; doi:10.1371/journal.pcbi.1005099)
Supplement: S1 Text — (PDF) [file pcbi.1005099.s001.pdf]

S1 Text: A statistical inference model

Uncertainty-dependent Extinction of Fear Memory in an Amygdala-mPFC Neural Circuit Model

Yuzhe Li[1], Ken Nakae[2], Shin Ishii[2] and Honda Naoki[3,*]
*: Corresponding author

1. Graduate School of Biostudies, Kyoto University, Kyoto, Japan
2. Graduate School of Informatics, Kyoto University, Kyoto, Japan
3. Imaging Platform of Spatio-temporal Information, Graduate School of Medicine, Kyoto University, Kyoto, Japan

Fear memory as a statistical inference

We aimed to reveal the type of statistical processing that animals perform during fear conditioning with partial reinforcement and subsequent extinction. The fact that extinction learning largely depends on the uncertainty of the US during fear conditioning (**Fig 2I-K**) suggests that the uncertainty must be encoded in the brain. Thus, animals may process the statistical properties of sequential US observations.

As statistical processing performed by the animals, we adopted a statistical model based on sequential updating of Bayesian logistic regression (see Statistical inference model below). Because continuous and discontinuous presentation of the US during full and partial reinforcement paradigms leads to fear memories that differ in their resistance to extinction, the effect of US continuity was incorporated into the model; the probability that the US would occur was inferred based on the previous observation of the US **(equation (S1)**). Then, our simulation demonstrated that, like the basic model, the sequentially predicted US probability reproduced, (**Fig 2**) the characteristics of the extinction of fear memory acquired through both the full and partial reinforcement schedules (**S3C Fig and S3D Fig)** in a manner consistent with biological fear neurons.

This statistical model also generated several properties that depended on the US probability, as obtained in the neural circuit model. These properties included the time constant of extinction (**S4B Fig**), the amount of residual fear memory that remained after extinction (**S4C Fig**), and the degree of surprise to the no-US (**S4D Fig**), which was quantified as the amount of information (**S3E Fig and S3F Fig**). Moreover, we compared the measure of surprise in the neural circuit model (the learning signals) with the measure of surprise in the statistical model (amount of information) when the same US pattern was applied, and we found a high degree of correlation, providing additional evidence that the learning signal in the neural circuit model represents the degree of surprise in terms of statistics **(S4E Fig and S4F Fig**). Taken together, these commonalities between the two models suggest that the neural circuit model that consisted of fear, persistent and extinction neurons effectively processed the statistical property of the occurrence of the US through sequential updating of Bayesian logistic regression.

Statistical inference model

To quantify the degree of surprise from the perspective of inferring whether the US would occur, we developed a statistical model based on logistic regression with sequential Bayesian updating [1]. We assumed that the animals inferred the probability that the US would occur based on the previous observation of the US as

$$\pi_t = \sigma(w_{t,0} + w_{t,1} US_{t-1}) , \qquad \text{(S1)}$$

and sequentially updated $\mathbf{w}_{t+1}=[w_{t+1,0}, w_{t+1,1}]$ to predict the probability based on a new instance of the US, where US_t denotes a binary variable, i.e., US=0 and =1 represent the presence and absence of the US, respectively; $w_{t,0}$ and $w_{t,1}$ are internal variables in animals, representing the effects of inferring the probability that the US would occur independent of and dependent on the previous US, respectively; $\sigma(\cdot)$ is the logistic function $\sigma(x)=1/(1+e^{-x})$; π_t represents the probability of the US occurrence that the animals infer based on the previous US, US_{t-1}. We also assumed that the degree of surprise (S) that animals feel in response to the US or no-US is quantified by the amount of information [2]:

$$\begin{aligned} &S(\text{US}) = -\log \pi_t \\ &S(\text{no-US}) = -\log(1-\pi_t) \end{aligned} \; . \qquad \text{(S2)}$$

We considered $\mathbf{w}_{t+1}$ to be updated by sequential Bayesian updating as

$$\begin{aligned} P(\mathbf{w}_t \mid US_{1:t}) &\propto P(US_t \mid \mathbf{w}_t, US_{1:t-1}) P(\mathbf{w}_t \mid US_{1:t-1}) \\ &= P(US_t \mid \mathbf{w}_t, US_{t-1}) \int P(\mathbf{w}_t \mid \mathbf{w}_{t-1}) P(\mathbf{w}_{t-1} \mid US_{1:t-1}) d\mathbf{w}_{t-1} \end{aligned} , \qquad \text{(S3)}$$

where $P(\mathbf{w}_t|\mathbf{US}_{1:t-1})$ and $P(\mathbf{w}_t|\mathbf{US}_{1:t})$ represent the prior and posterior distributions, respectively. The animals were assumed to think that instances of the US were simply generated by a Bernoulli process as

$$P(US_t \mid \mathbf{w}_t, US_{t-1}) = \pi_t^{US_t}(1-\pi_t)^{1-US_t} . \qquad \text{(S4)}$$

In addition, the animals were assumed to believe that $\mathbf{w}_t$ remained almost constant with a small degree of noise as $\mathbf{w}_t=\mathbf{w}_{t-1}+\boldsymbol{\varepsilon}_t$, where $\boldsymbol{\varepsilon}_t$ represents independent and identically distributed Gaussian noise with a mean of zero and a low variance, s. Then,

$$P(\mathbf{w}_t \mid \mathbf{w}_{t-1}) = N(\mathbf{w}_t \mid \mathbf{w}_{t-1}, s\mathbf{I}) , \qquad \text{(S5)}$$

where $\mathbf{I}$ represents the unit matrix.

The Bayesian belief update was implemented as an extended Kalman filter, in which the prior and posterior distributions are approximated by Gaussian distributions. $P(\mathbf{w}_{t-1}|\mathbf{US}_{1:t-1})$ was represented by

$$P(\mathbf{w}_{t-1} \mid US_{1:t-1}) = N(\mathbf{w}_{t-1} \mid \mu_{t-1}, \Sigma_{t-1}) , \qquad \text{(S6)}$$

where N represents the function of the Gaussian distribution parameterized by $\boldsymbol{\Sigma}_t$ and μ_t, which are the variance-covariance matrix and mean vector of $\mathbf{w}_t$, respectively. Then, equation (S3) can be transformed into

$$P(\mathbf{w}_t \mid US_{1:t}) \propto P(US_t \mid \mathbf{w}_t, US_{1:t-1}) N(\mathbf{w}_t \mid \mu_{t-1}, s + \Sigma_{t-1}) . \qquad \text{(S7)}$$

Note that the prior and posterior distributions in equation (S7) do not have conjugate relationship. Thus, the posterior distribution, $P(\mathbf{w}_t|US_{1:t})$, was approximated by a Gaussian distribution using

Laplace approximation as

$$P(\mathbf{w}_t \mid US_{1:t}) = N(\mathbf{w}_t \mid \mu_t, \Sigma_t) , \quad \text{(S8)}$$

where μ_t and Σ_t are updated as

$$\mu_t = \arg\max_{\mathbf{w}_t} E(\mathbf{w}_t) , \quad \text{(S9)}$$

$$\Sigma_t^{-1} = -\nabla\nabla E(\mathbf{w}_t) = (s + \Sigma_{t-1})^{-1} + \sigma(\mu_t^{\mathrm{T}}\phi_{t-1})(1 - \sigma(\mu_t^{\mathrm{T}}\phi_{t-1}))\phi_{t-1}\phi_{t-1}^{\mathrm{T}} , \quad \text{(S10)}$$

where ϕ indicates $(1, US_{t-1})^{\mathrm{T}}$ and

$$\begin{aligned} E(\mathbf{w}_t) &= -\frac{1}{2}(\mathbf{w}_t - \mu_{t-1})^{\mathrm{T}}(s + \Sigma_{t-1})^{-1}(\mathbf{w}_t - \mu_{t-1}) \\ &\quad + US_t \log\sigma(\mathbf{w}_t^{\mathrm{T}}\phi_{t-1}) + (1 - US_t)\log(1 - \sigma(\mathbf{w}_t^{\mathrm{T}}\phi_{t-1})) + const \end{aligned} . \quad \text{(S11)}$$

To calculate μ_t requires numerical optimization because $E(\mathbf{w}_t)$ is a non-linear function of $\mathbf{w}_t$.

References

1. Ng AY, Jordan MI. On discriminative vs. generative classifiers: a comparison of logistic regression and naive Bayes. In: Dietterich TG, Becker S, Ghahramani Z, editors. Advances in neural information processing systems 14, Vol. 2. Cambridge, MA: MIT Press; 2002. pp. 841-848.
2. Shannon CE. A mathematical theory of communication. Bell Syst Tech J. 1948;27: 379–423. doi:10.1002/j.1538-7305.1948.tb01338.x
