# Supplementary figures and images for: Uncertainty-Dependent Extinction of Fear Memory in an Amygdala-mPFC Neural Circuit Model
Source: PLoS Comput Biol. 2016 Sep 12;12(9):e1005099. doi: 10.1371/journal.pcbi.1005099 (PMC5019407; doi:10.1371/journal.pcbi.1005099)

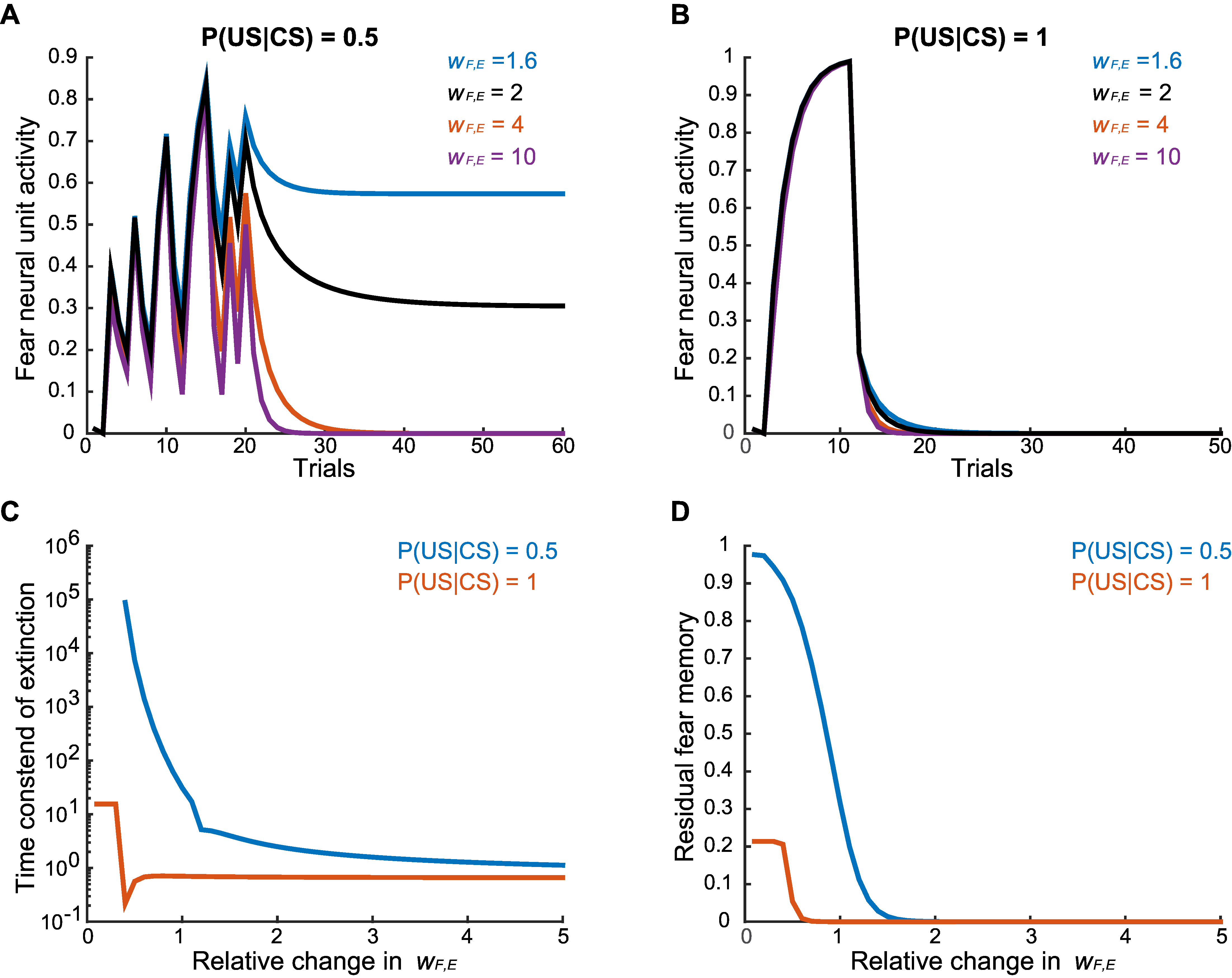

Supplement: S1 Fig — (A) Fear neural unit activity during the partial reinforcement fear conditioning (P(US|CS) = 0.5) and subsequent extinction was shown with various changes in wF,E. (B) Fear neural unit activity during the full reinforcement fear conditioning (P(US|CS) = 1) and subsequent extinction was shown with various changes in wF,E. Note that αE was also concurrently changed such that wF,E αE = const. (C) Red and blue lines indicate the time constant of extinction after the full and partial reinforcement fear conditioning, respectively, varying wF,E. The time constant of extinction is defined in the legend of Fig 2. (D) Red and blue lines indicate the residual activity of the fear neural unit after the extinction following the full and partial reinforcement fear conditioning, respectively, varying wF,E. (TIF) [file pcbi.1005099.s002.tif]

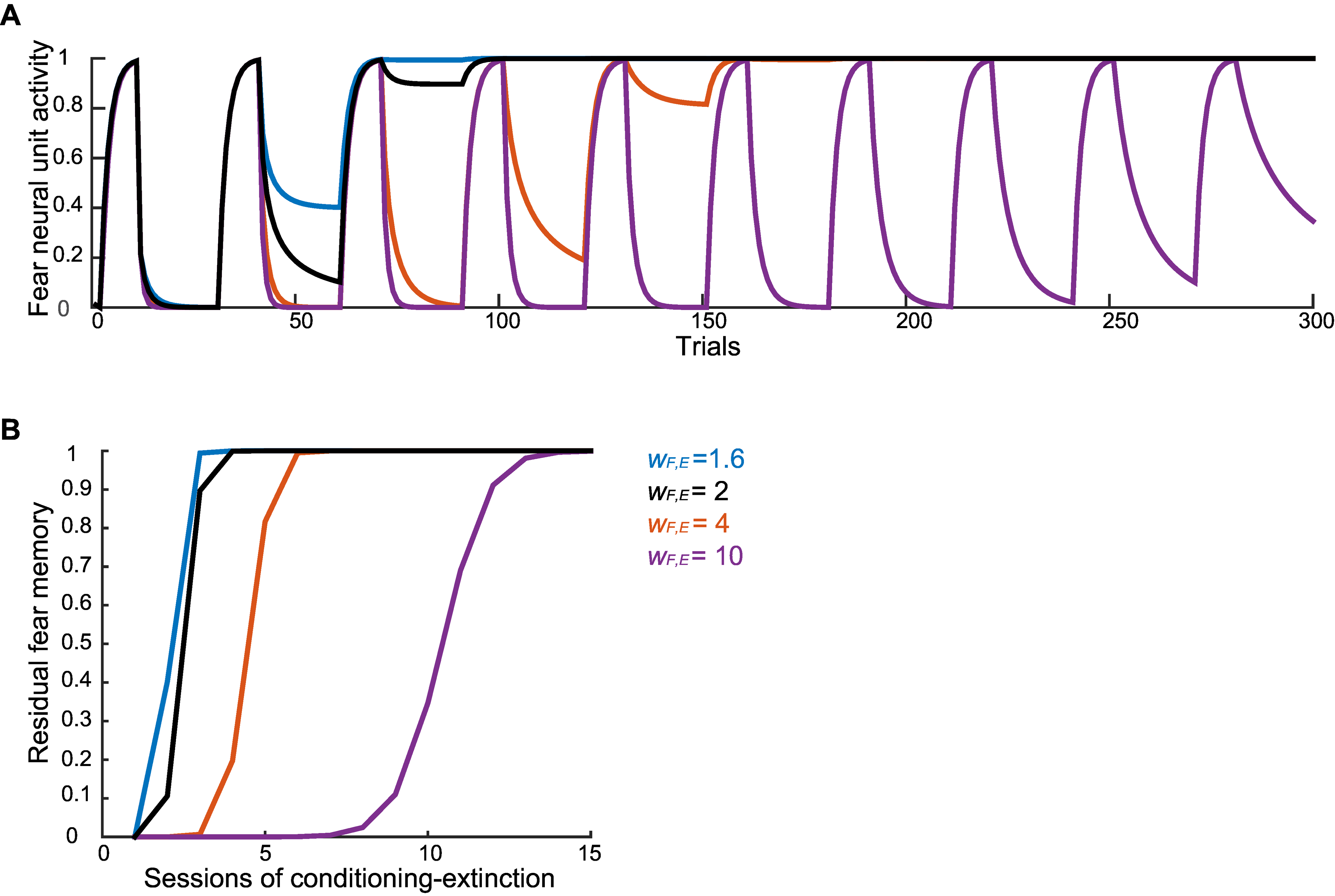

Supplement: S2 Fig — (A) The fear neural unit activity during successive conditioning and extinction with changes in wF,E. Note that αE was concurrently changed such that wF,E αE = const. (B) The residual fear memory after each extinction session was plotted with the change in wF,E. (TIF) [file pcbi.1005099.s003.tif]

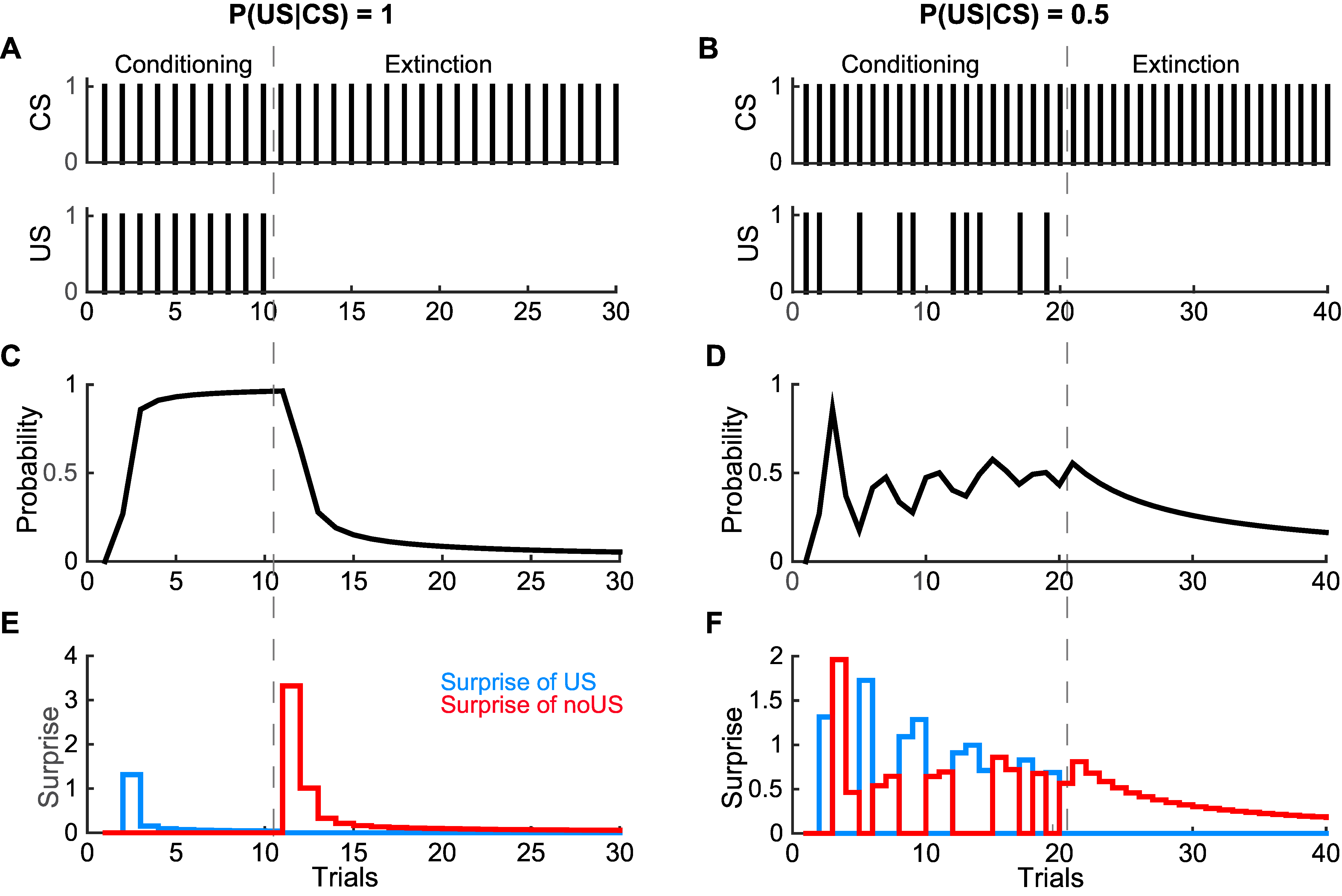

Supplement: S3 Fig — Simulation results for the statistical inference model with full and partial reinforcement schedules are presented in the left (A, C, and E) and right (B, D, and F) columns, respectively. (A, B) The CS and US events were applied according to the same schedule shown in Fig 2A and 2B. (C, D) The black lines indicate the US probability estimated by logistic regression with sequential Bayesian updating. (E, F) The blue and red lines indicate the degree of surprise for the US and no-US, which is measured as the amount of information and calculated as −logP(US) and −log(1−P(US)), respectively. (TIF) [file pcbi.1005099.s004.tif]

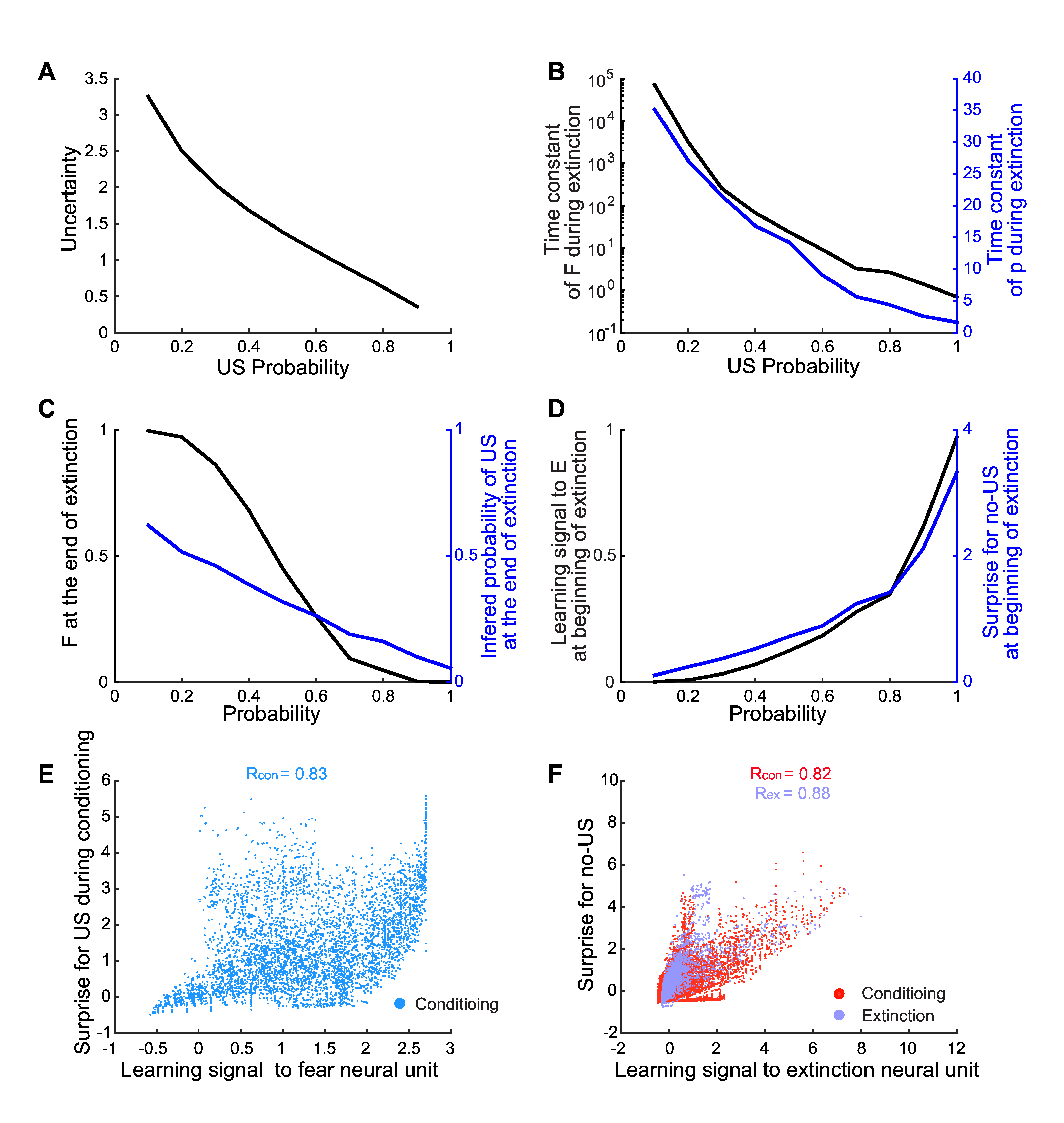

Supplement: S4 Fig — (A) The black line indicates the uncertainty of the next US observation as a function of the probability of the US. (B) The black and blue lines indicate the time constant of fear memory decline during extinction as a function of the probability that the US will be presented during fear conditioning in the basic neural circuit model and the statistical inference model, respectively. (C) The black and blue lines indicate the fear memory at the end of extinction as a function of US probability during fear conditioning in the basic neural circuit model and the statistical inference model, respectively. (D) The black and blue lines indicate the surprise associated with the no-US, i.e., the learning signal to the extinction neural unit in the basic neural circuit model and the amount of information associated with a no-US observation in the statistical inference model, respectively, as a function of US probability during fear conditioning. (E, F) Comparison between learning signals in the basic neural circuit model and surprise in the statistical inference model when the same US schedule was applied in both models. Each dot in (E) represents the relationship between ‘learning signals to CS-related synaptic inputs to fear and persistent neural units’ and ‘surprise for US’ during fear conditioning (blue dots) at each trial, and each dot in (F) represents the relationship between ‘learning signals to CS-related synaptic inputs to the extinction neural unit’ and ‘surprise for no-US’ during fear conditioning (red dots) and extinction (magenta dots) at each trial. Note that during extinction, the surprise associated with the US and the learning signal to the fear neural unit are both 0 due to the absence of US input. Therefore, this relationship is not included in (E). (TIF) [file pcbi.1005099.s005.tif]

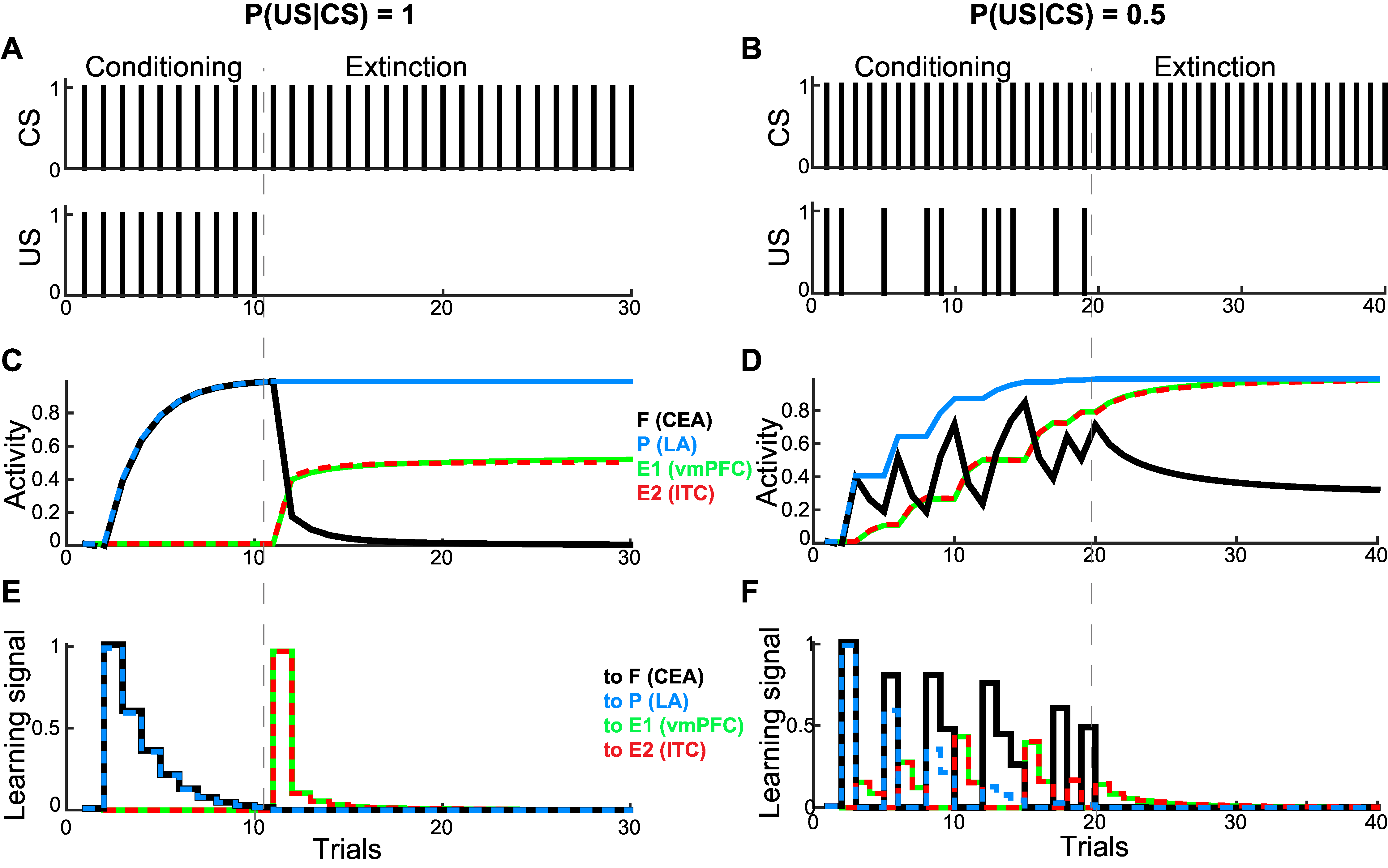

Supplement: S5 Fig — Simulation results for the extended model with full and partial reinforcement schedules are presented in the left (A, C and E) and right (B, D and F) columns, respectively. (A, B) CS and US schedules during fear conditioning and extinction. (C, D) The blue, green, red and black lines represent the activity of the LA (persistent neurons), vmPFC (extinction neurons), ITC (another group of extinction neurons) and CEA (fear neurons), respectively. Note that the green lines are almost invisible because they overlap with the red lines. (E, F) The blue, green, red and black lines represent the learning signals that change the weights of CS-related synaptic inputs to the LA, vmPFC, ITC and CEA, respectively. Note that the blue and green lines are almost invisible because they overlap with the black and red lines, respectively. Note that each panel looks the same as Fig 2A–2D, 2G and 2H, which correspond to the basic model, because the same values were used for the parameters (S1 Table). (TIF) [file pcbi.1005099.s006.tif]

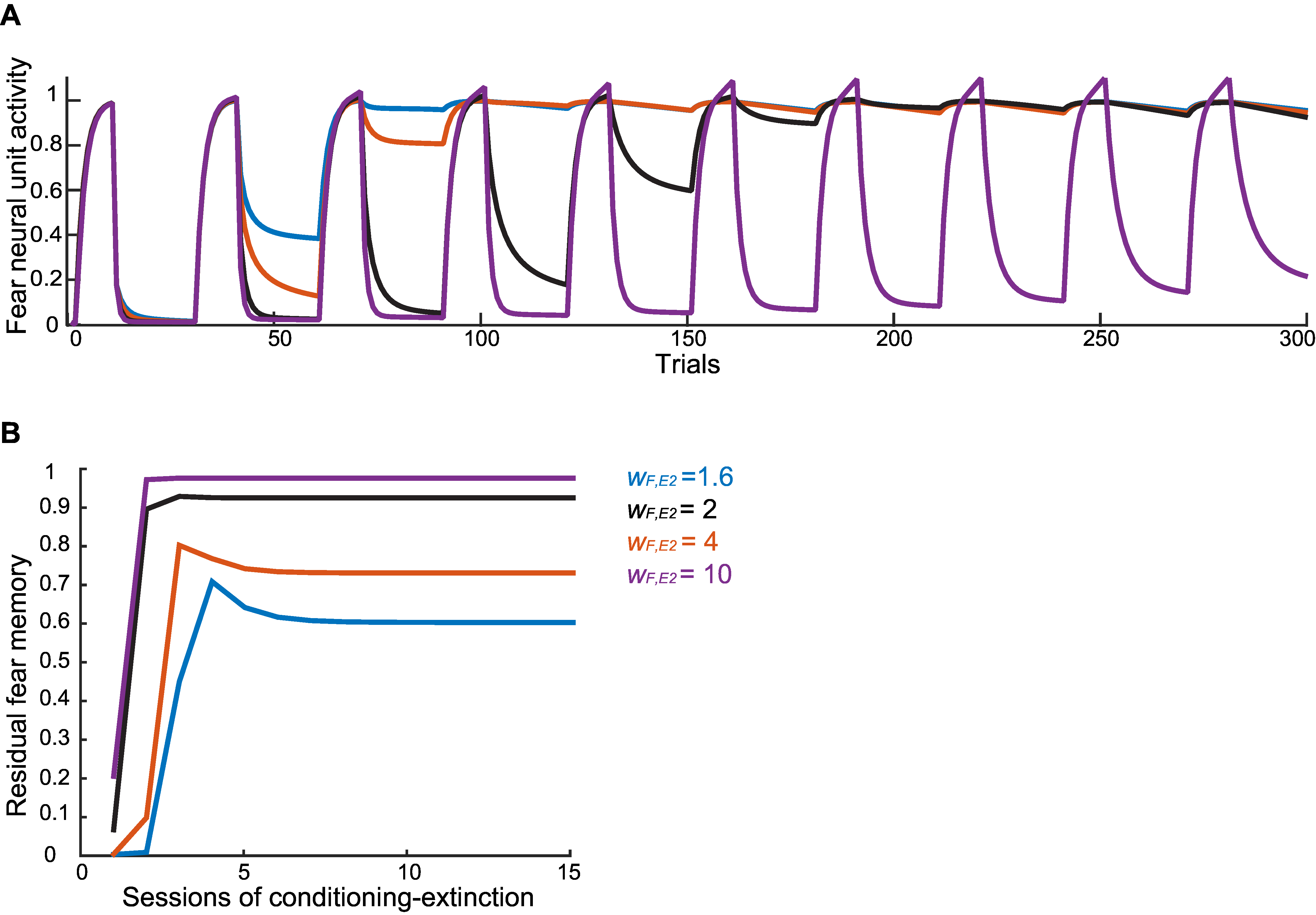

Supplement: S6 Fig — (A) The fear neural unit activity during successive conditioning and extinction with changes in wF,E2. Note that αE2 was concurrently changed such that wF,E2 αE2 = const. (B) The residual fear memory after each extinction session was plotted against the change in wF,E2. (TIF) [file pcbi.1005099.s007.tif]

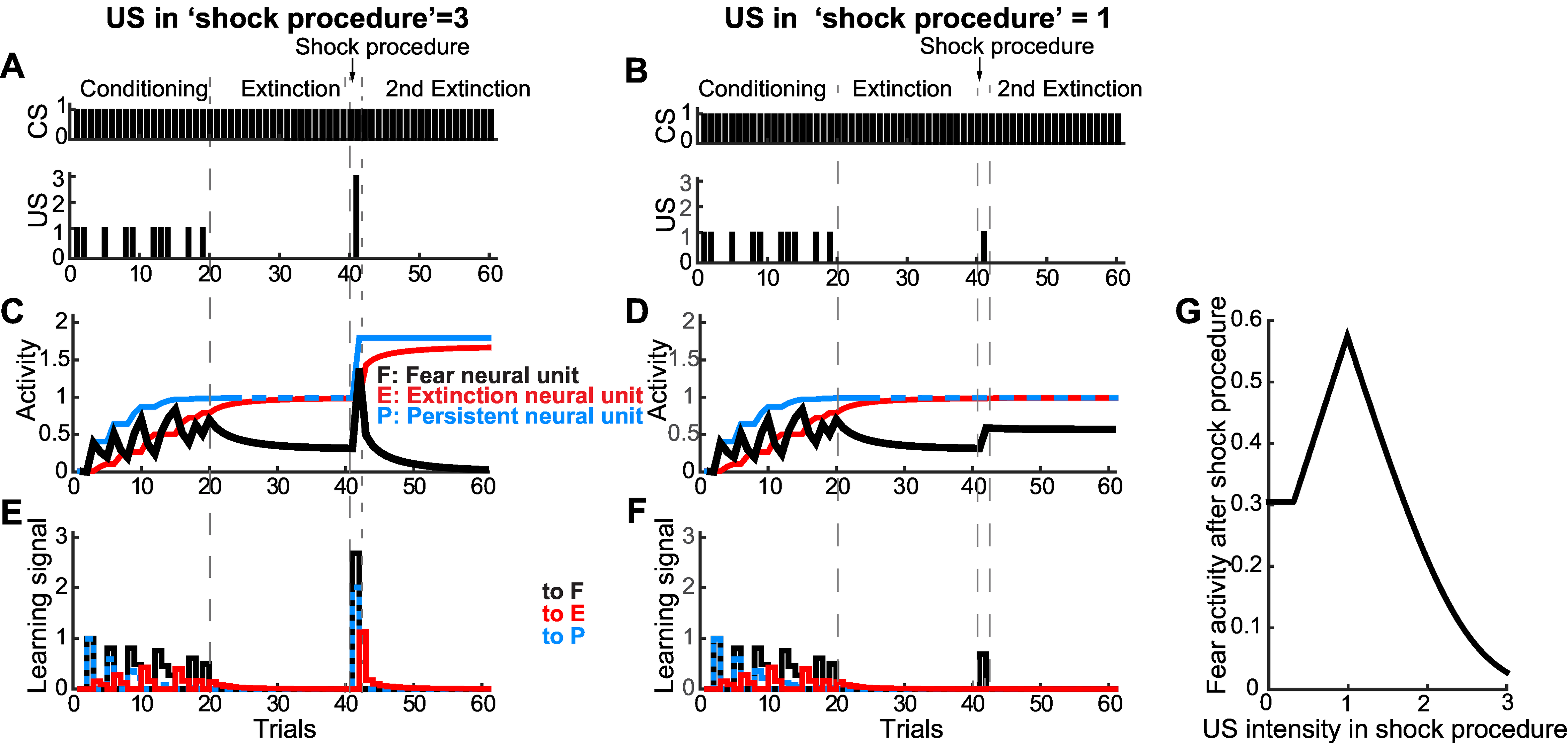

Supplement: S7 Fig — (A, B) CS and US schedules; after the extinction training for the partially reinforced fear memory, an additional CS-US pairing was applied, in which the US was three times stronger (A) or the same intensity (B). (C, D) The black, blue and red lines represent the activity of the fear, persistent and extinction neural units, respectively. (E, F) The black, blue and red lines represent the learning signals to the fear, persistent and extinction neural units, respectively. (G) Effect of US intensity on the effectiveness of the shock procedure. Note that each panel looks the same as those in Fig 4, which corresponds to the extended model, because the same values were used for the parameters (S1 Table). (TIF) [file pcbi.1005099.s008.tif]

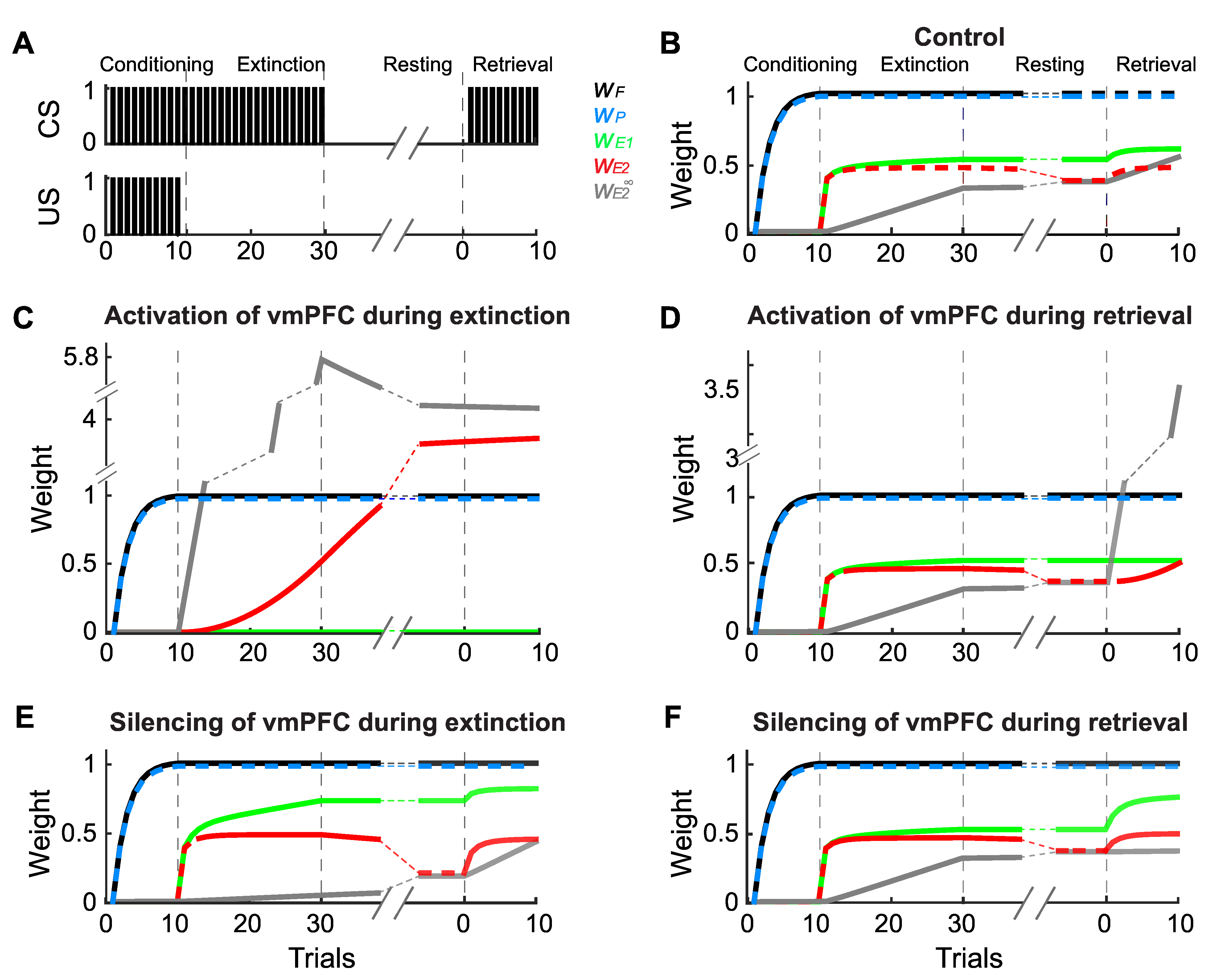

Supplement: S8 Fig — (A) The same as Fig 3A. (B-F) Changes in the synaptic weights in Fig 3. The blue, green, red, and black lines represent the early-phase plasticity-regulated weight of CS-related synapses to the LA (persistent neurons), vmPFC (extinction neurons), ITC (another group of extinction neurons) and CEA (fear neurons), respectively, and the grey lines represent the late-phase plasticity-regulated weight of CS-related synapses to the ITC. (TIF) [file pcbi.1005099.s009.tif]

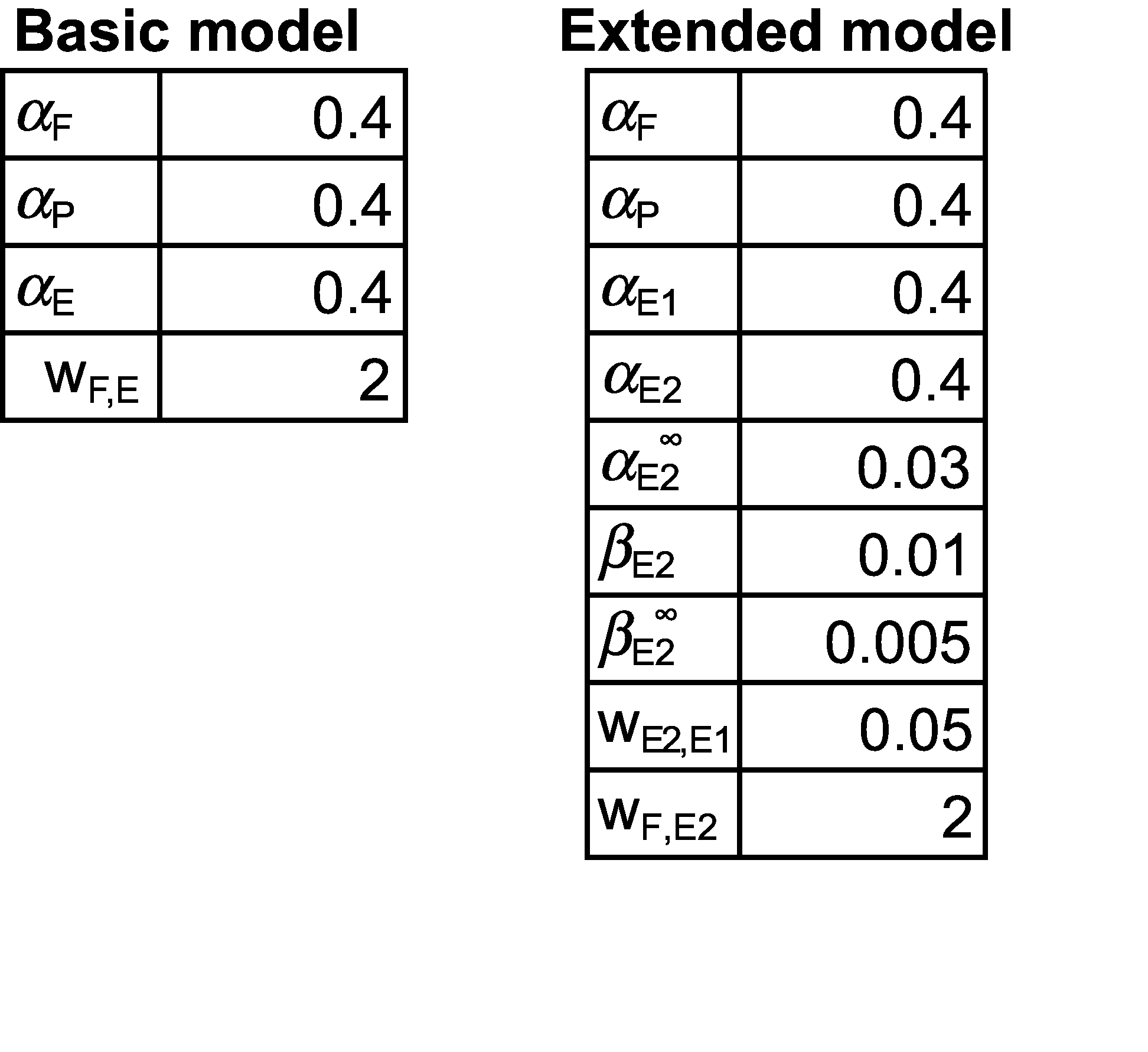

Supplement: S1 Table — (TIF) [file pcbi.1005099.s010.tif]
